# CDK4/6 inhibition synergizes with inhibition of P21-Activated Kinases (PAKs) in lung cancer cell lines

**DOI:** 10.1371/journal.pone.0252927

**Published:** 2021-06-17

**Authors:** Gabriela M. Wright, Nick T. Gimbrone, Bhaswati Sarcar, Trent R. Percy, Edna R. Gordián, Fumi Kinose, Natália J. Sumi, Uwe Rix, W. Douglas Cress

**Affiliations:** 1 Department of Molecular Oncology, Moffitt Cancer Center and Research Institute, Tampa, Florida, United States of America; 2 Department of Thoracic Oncology, Moffitt Cancer Center and Research Institute, Tampa, Florida, United States of America; 3 Department of Drug Discovery, Moffitt Cancer Center and Research Institute, Tampa, Florida, United States of America; 4 Cancer Biology PhD Program, University of South Florida, Tampa, Florida, United States of America; Columbia University, UNITED STATES

## Abstract

Theoretically, small molecule CDK4/6 inhibitors (CDK4/6is) represent a logical therapeutic option in non-small cell lung cancers since most of these malignancies have wildtype RB, the key target of CDKs and master regulator of the cell cycle. Unfortunately, CDK4/6is are found to have limited clinical activity as single agents in non-small cell lung cancer. To address this problem and to identify effective CDK4/6i combinations, we screened a library of targeted agents for efficacy in four non-small cell lung cancer lines treated with CDK4/6 inhibitors Palbociclib or Abemaciclib. The pan-PAK (p21-activated kinase) inhibitor PF03758309 emerged as a promising candidate with viability ratios indicating synergy in all 4 cell lines and for both CDK4/6is. It is noteworthy that the PAKs are downstream effectors of small GTPases Rac1 and Cdc42 and are overexpressed in a wide variety of cancers. Individually the compounds primarily induced cell cycle arrest; however, the synergistic combination induced apoptosis, accounting for the synergy. Surprisingly, while the pan-PAK inhibitor PF03758309 synergizes with CDK4/6is, no synergy occurs with group I PAK inhibitors FRAX486 or FRAX597. Cell lines treated only with Ribociclib, FRAX486 or FRAX597 underwent G1/G0 arrest, whereas combination treatment with these compounds predominantly resulted in autophagy. Combining high concentrations of FRAX486, which weakly inhibits PAK4, and Ribociclib, mimics the autophagy and apoptotic effect of PF03758309 combined with Ribociclib. FRAX597, a PAKi that does not inhibit PAK4 did not reduce autophagy in combination with Ribociclib. Our results suggest that a unique combination of PAKs plays a crucial role in the synergy of PAK inhibitors with CDK4/6i. Targeting this unique PAK combination, could greatly improve the efficacy of CDK4/6i and broaden the spectrum of cancer treatment.

## Introduction

Loss of cell cycle control represents one of the hallmarks of cancer. The well described Rb/E2F/CDK pathway is a driver in multiple cancers and parts of it can be found to be disrupted in most cancer types. However, targeting the E2F proteins has shown low efficacy [1–3]. Alternative to E2F inhibition, CDK inhibitors (CDKis) can be used to target the cell cycle. Several generations of CDKis have been developed over the last 20 years. Some inhibitors such as dinaciclib showed promising pre-clinical data but clinical trials in many tumors did not live up to the promise [4]. In the last ten years the need for high specificity was recognized. Three CDK4/6 inhibitors (CDK4/6is) Palbociclib, Ribociclib (LEE011) and Abemaciclib have been used in a variety of clinical trials in breast cancer [5–9]. By themselves, the CDK4/6is have some efficacy in breast cancer, but they are most effective when used in combination with estrogen receptor inhibitors and aromatase inhibitors. Its effect on the cell cycle makes CDK4/6 good candidate targets in almost any cancer type. For CDK4/6is to be effective, Retinoblastoma (Rb) must be intact. In non-small cell lung cancer (NSCLC), Rb is mutated in only 15% of cases. Silencing of the E2F/Rb/CDK pathway can occur via other components of the pathway, such as p16 silencing (40–70%), which, when active, inhibits CDK4/6. With p16 silenced, CDK4/6 phosphorylates and inactivates Rb and allows cells to proceed into the cell cycle. Like many targeted compounds used in cancer, CDK4/6is have not shown broad efficacy as single therapeutic agents. Work in the clinic with various targeted therapies has shown that single agents can have strong initial responses, but in addition to the occurrence of initial primary resistance, acquired resistance seems inevitable [10]. Preclinical models looking for synergy have shown positive effects combining CDK4/6i with inhibitors targeting molecules such as EGFR, MEK, mTOR or BRAF V600E in various cancers, as well as the ability to overcome resistance [11–14]. Analogous to synthetic lethality, we could potentially broaden the clinical efficacy of CDK4/6i and identify compounds that would synergize with them in lung cancer cell lines. Using combination therapies may, for both types of resistance, be a more effective way to gain the upper hand over the disease. We hypothesize, that the utility of CDK4/6i could be broadened if drug targeting synergistic pathways were known.

Here we describe the discovery of synergy between the pan-PAK inhibitor PF03758309 and the CDK4/6i Ribociclib in NSCLC cell lines and investigate the mechanism behind this synergy. P21-activated kinases (PAKs) are a family of small GTPase effectors, specifically of Rac and CDC42. They are divided into 2 groups, Group I (PAK1-3) and Group II (PAK4-6) based on chemical and structural properties, as well as the regulation of their activation [15–17]. PAKs play a critical role in cancer progression as they are at the nexus of many pathways, including cytoskeletal remodeling (affecting shape and motility), cell cycle progression and cell survival (proliferation and apoptosis), processes essential for cancer development and metastases. PAKs have been shown to promote anchorage independent growth and survival by inhibiting tumor suppressors such as Merlin (by PAK2) [18,19], pro-apoptotic factors such as BAD (by PAK1, 4 and 5) [20–22] or activating anti-apoptotic factors such as Raf and MEK1 [23]. PAK2 can act both pro- and anti-apoptotic [24,25]. PAK3 has previously been shown to be able to compensate for PAK1 in a KRAS skin cancer model system [26].

Both ATP competitive and allosteric inhibitors have been developed. Several ATP-competitive PAK inhibitors are available, such as the compound identified in our screen. PF03758309 was one of the first PAKis. It was initially designed as a PAK4i but has broad activity against all PAKs. Clinical trials with this compound were terminated due to lack of bioavailability, and thus, lack of tumor response [27], but the drug is still a useful tool compound. Another ATP competitive inhibitor in our custom compound library, FRAX486, has strong activity against PAK1-3, and although some activity was detected against PAK4 it was much less potent [28,29]. Our previous work has shown that PAK1 (p21-activated kinase 1) is regulated by Rb [30]. Analysis of the PAK6 promotor sequence shows binding sites for E2F suggesting regulation by Rb as well [31]. This implies that CDK4/6is could affect these PAKs even without the use of PAK inhibitors. Since PAKs are involved in so many cellular processes, including cell cycle, discovering the mechanism behind the synergy could contribute to a wider knowledge of PAK interactions as well as provide new options for treatments of NSCLC. Here, we discovered a new synergistic interaction between CDK4/6is and PAKi in NSCLC, and mechanistically show that through the inhibition of multiple PAKs autophagy is reduced allowing for apoptosis to achieve this synergy.

## Results

### PF03758309 is synergistic with CDK4/6i

**Fig 1:**
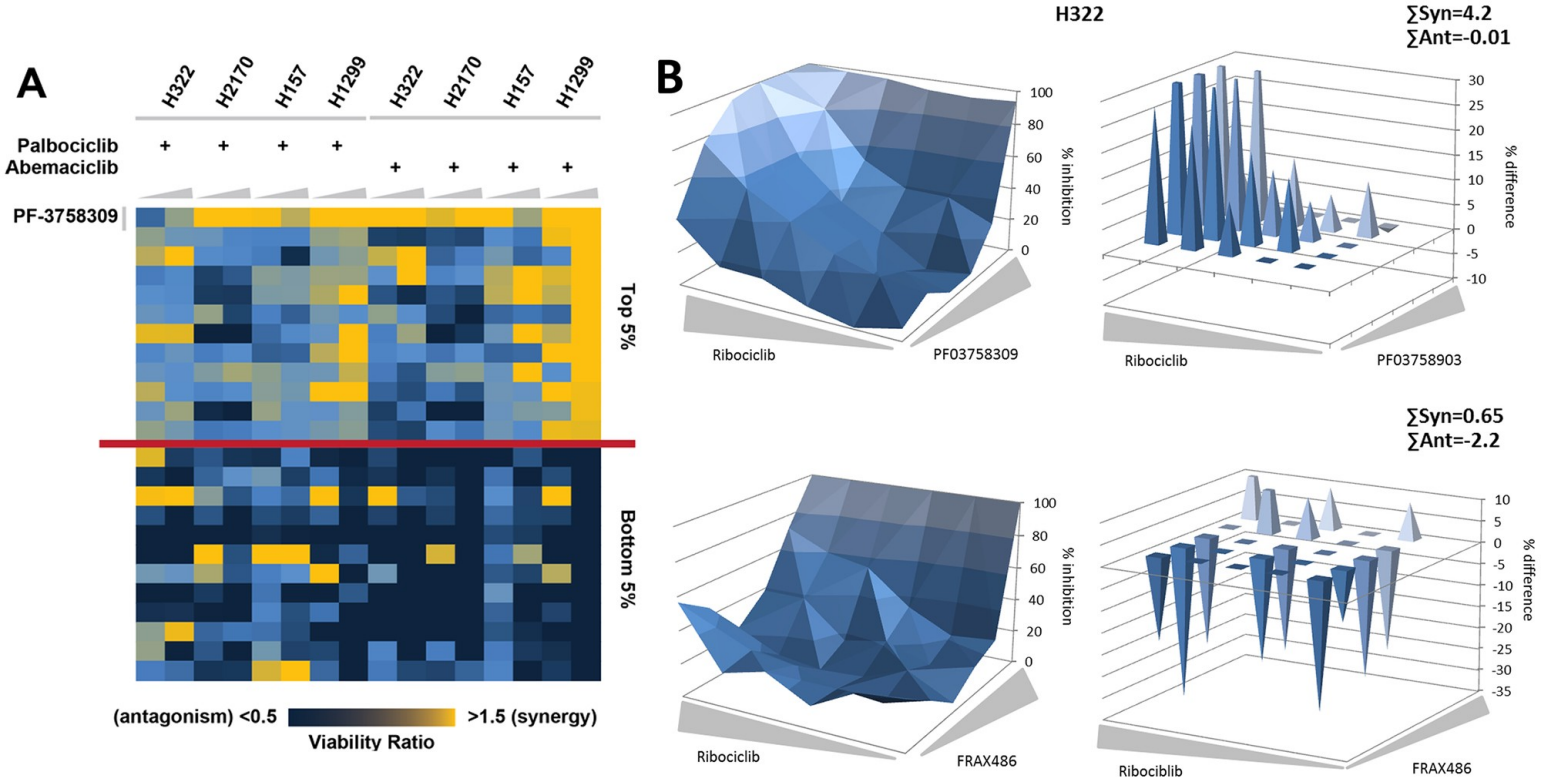
Combination drug effects of clinical CDK4/6is in lung cancer cells. **A)** Drug combination screening with a customized library of 240 targeted agents (0.5 μM and 2.5 μM, increasing wedge size) and either palbociclib (PD-0332991–2.5 μM) or abemaciclib (LY2835219–0.75 μM), depicted as a heat map, suggests synergy particularly with the PAK inhibitor PF03758309. **B)** Validation of drug synergy. Left panel: full dose-response matrix depicting inhibition of viability as determined using a CTG viability assay with varying drug concentrations (Ribociclib: 0–20 μM; PF03758309 or FRAX486: 0–10 μM). Right panel: deviation from calculated Bliss value for additivity (in % viability difference); needles pointing up indicate synergy, needles pointing down antagonism.

To identify drugs synergistic with CDK4/6is, we performed a drug combination screening in 4 NSCLC cell lines (H157, H332, H1299 and H2170; with a customized library of 240 targeted agents in combination with palbociclib or abemaciclib. S1 Table provides a description of the cell lines used and S2 Table provides the library screening results. Out of all the treatments, the pan-PAK inhibitor PF03758309 rose to the top with viability ratios indicating synergy in all 4 cell lines and for both CDK4/6is (Fig 1A). To validate the drug screening, a full dose-response matrix was generated for each cell line showing inhibition of viability, with varying combinations of drug concentrations, as measured by CTG assay. The synergy between the CDK4/6i Ribociclib and PF03758309 was determined using the Bliss model of independence as shown for cell lines H322 (Fig 1B and Table 1), H157, H1299 and H2170 (Table 1 and S1A Fig). Synergy with the CDK4/6is used in the screen, Abemaciclib and Palbociclib, were validated as well (Table 1). FRAX486, a PAKi that targets only a subset of the PAK family, was included in the screening and validation, (Figs 1B and S2, Tables 1 and 2). In the screening, FRAX486 did not show an altered ratio, suggesting lack of synergy. Although not included in the screening, synergy with Ribociclib was tested for another partial PAKi, FRAX597 (Table 2 and S3 Fig). The results of the validation show strong synergy between CDK4/6is and PF3758309, but much less so for the combination with FRAX486, consistent with the screening data. No synergy was observed for the combination with FRAX597 (Σ synergy: H322, 0.1 ± 0.06; H2170, 0.13 + 0.07; H322 and H1299 both 0). Individual EC50 values were determined from the synergy matrix (S4 Fig). In addition to the cell lines discussed above, synergy between PF03758309 and Ribociclib was observed in 6 more lung cancer cell lines and, as expected, in an pRb null lung cancer cell line (H2172). No synergy was observed in HBEC, an immortalized human lung bronchial epithelial cell line (S1B Fig). Drug activity was confirmed by the lack of phosphorylation of the PAK1 and PAK4 target, BAD, at serine 112 (S5 Fig).

**Table 1:** Synergy values.

	PF03758309 (n = 3–8)				FRAX486 (n = 1–5)			
cell line	H157	H322	H2170	H1299	H157	H322	H2170	H1299
**Abemaciclib**	**3.58** ± **0.50**	**4.09** ± **0.71**	**2.09** ± **0.22**	**nt**	**0.39** ± **0.26**	**0.37**	**0.46**	**nt**
**Palbociclib**	**2.05** ± **0.48**	**2.83** ± **0.13**	**1.78** ± **0.24**	**nt**	**0.37** ± **0.12**	**0.55**	**0.23**	**nt**
**Ribociclib**	**2.20** ± **0.33**	**2.85** ± **0.41**	**1.70** ± **0.15**	**0.58** ± **0.05**	**0.32** ± **0.09**	**0.61** ± **0.28**	**1.02** ± **0.23**	**0.15** ± **0.09**

Synergy values (∑synergy ± standard error) for Abemaciclib, Palbociclib or Ribociclib combined with PF03758309 or FRAX486, in four cell lines, H157, H322, H1299 and H2170 (nt–not tested).

**Table 2:** In vitro IC_50_ and Ki values of PF03758309, FRAX486 and FRAX597.

	PF03758309^a^		FRAX486		FRAX597
	IC50	Ki	IC50^b^	IC50^c^	IC50^d^
PAK1		13.7 nM	8.25 nM	14 nM	8 nM
PAK2	190 nM		39.5 nM	33 nM	13 nM
PAK3	99 nM		55.3 nM	39 nM	19 nM
PAK4	1.3 nM	18.7 nM	779 nM	575 nM	
PAK5		18.1 nM			
PAK6		17.1 nM			

*In vitro* IC_50_ and Ki values of PF03758309, FRAX486 and FRAX597. Both measurements indicate concentration at which ½ max inhibition occurs *in vitro*.

Data are reported in three references,

^a^ [32],

^b^ [28],

^c^ [29],

^d^ [33].

### PF03758309 causes G2 cell cycle arrest

**Fig 2:**
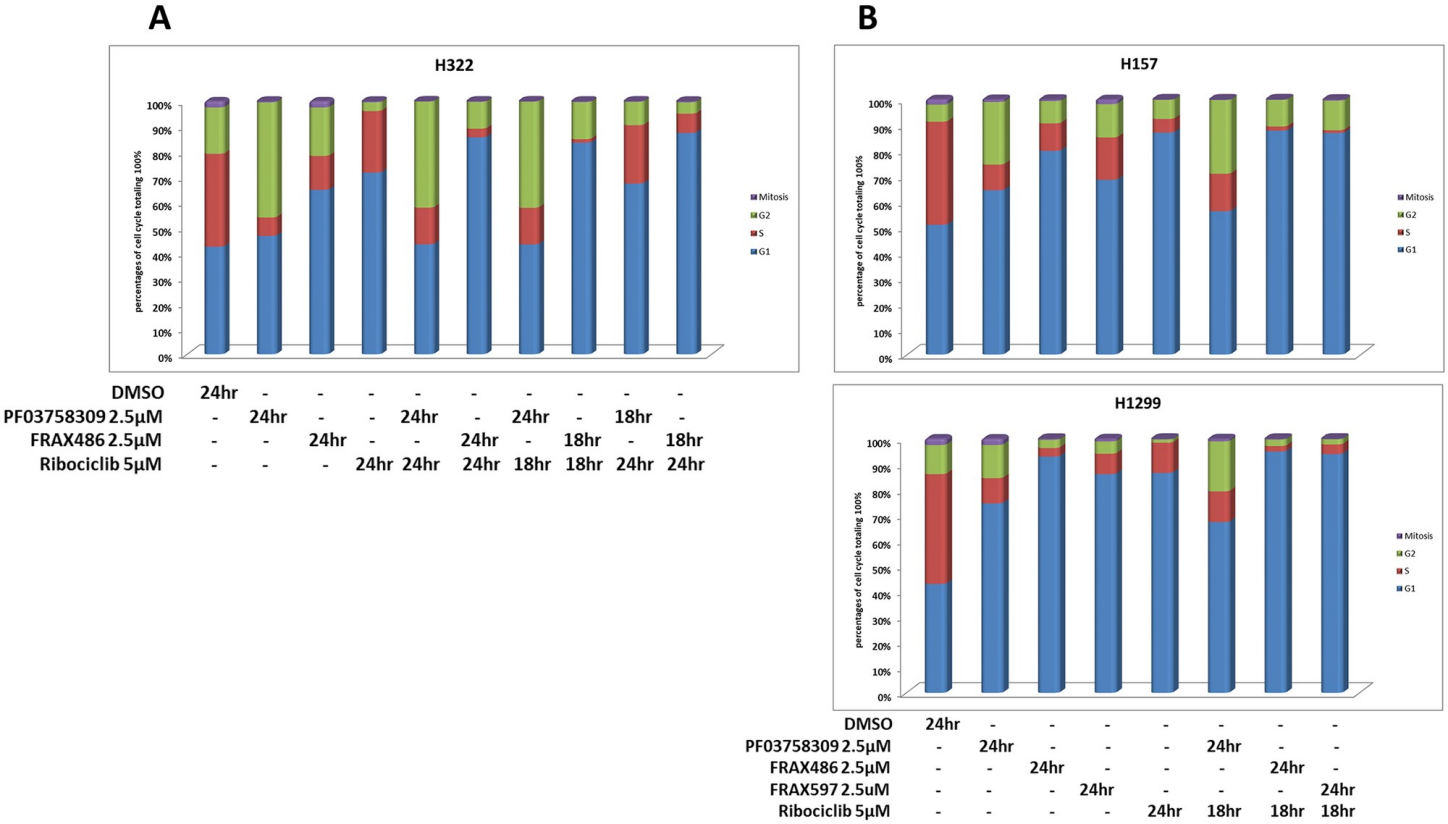
Cell cycle arrest and apoptosis. **A**. Percentage of H322 cells in various stages of the cell cycle after 24 hours for single drug (PF03758309, FRAX486 and Ribociclib) or drug combination, as well as with consecutive drug addition at 24 hours and 18 hours before harvest. **B**. Percentage of H157 or H1299 cells in various stages of the cell cycle after 24 hours for single drug or combination drug with PAKi (including FRAX597), added for 24 hours, and Ribociclib for 18 hours.

To determine the role cell cycle arrest plays in the observed viability reduction and synergy, cell cycle progression was determined by FLOW cytometry in H322 for single agent, simultaneously administered combination of agents, or administered 6 hours apart (either Ribociclib first or PAKi first) using DAPI and pSer10 Histone H3 (pHH3), the last a marker of mitosis. As seen in Fig 2A, Ribociclib resulted in G0/G1 arrest, as expected, while PF03758309 yielded a G0/G1 arrest as well as a G2 arrest. FRAX486, like Ribociclib, appeared to have a G0/G1 arrest only. The combination with PF03758309 reduced the G0/G1 arrest of Ribociclib and resulted in both a G0/G1 and G2 arrest similar to that seen by PF03758309 alone. FRAX486 on the other hand appears to have no effect on the G0/G1 arrest of Ribociclib and if anything, adds to the effect (Fig 2A). The G2 arrest is what sets PF03758309 apart from FRAX486. This G2 arrest is completely absent when Ribociclib is administered first. To determine if this observation is unique to H322, we examined additional cell lines: H157 and H1299 were examined for relevant conditions, and for the new cell lines FRAX597 was added as a third PAKi (Fig 2B). Results show a similar trend in both lines toward a G1/G0 and G2 arrest with PF03758309, not seen for FRAX486 or FRAX597.

### PF03758309 alone and in combination with Ribociclib causes apoptosis

**Fig 3:**
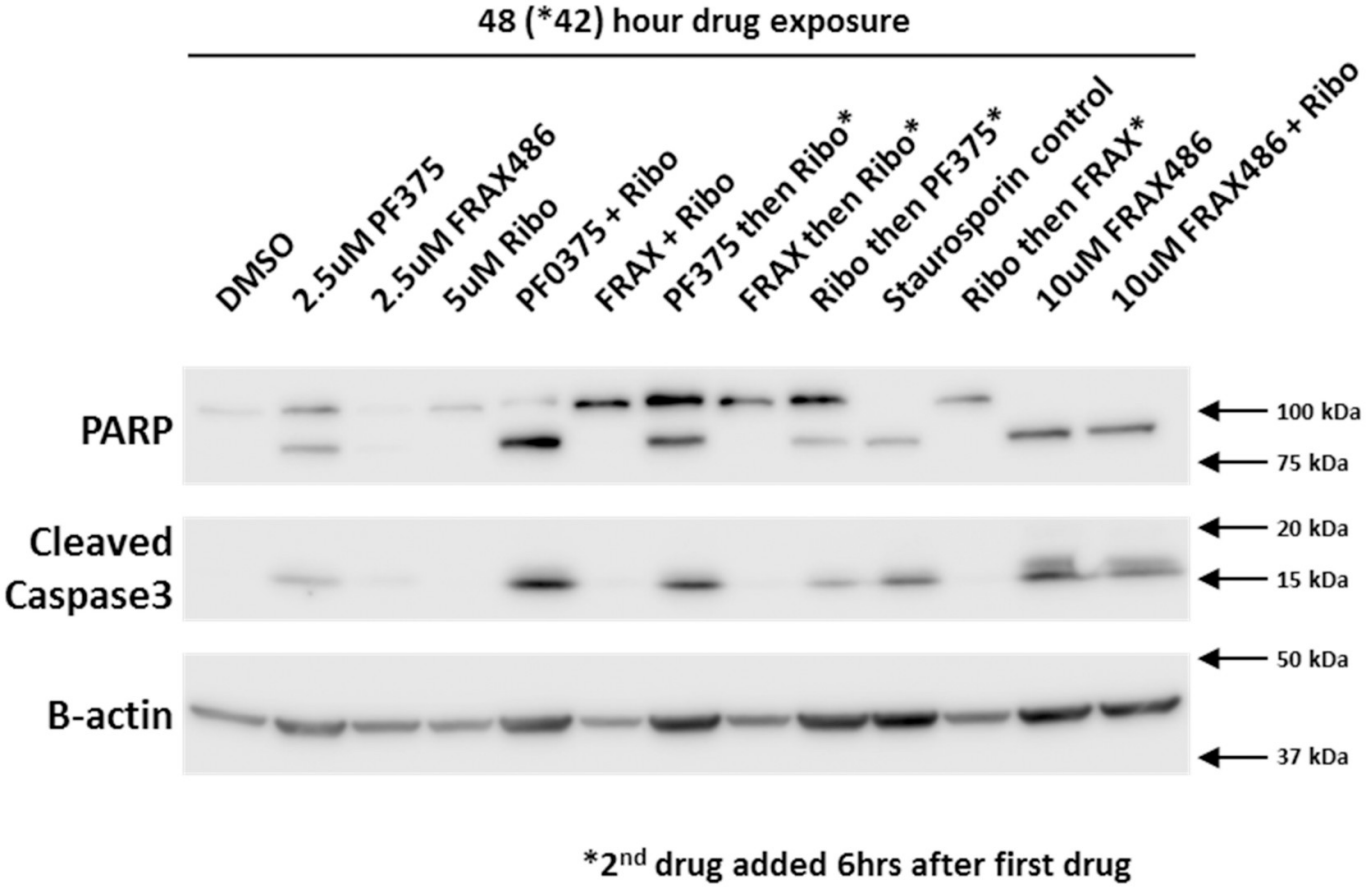
Cleavage of Caspase 3 and PARP following treatment with PF03758309 and its combinations. Apoptosis was determined by immunoblot detection of cleaved Caspase 3 and PARP measured in H322 after 48 hours treatment with the indicated drugs. Drug concentrations were the same as in Fig 2 with the addition of 10μM FRAX486.

**Fig 4:**
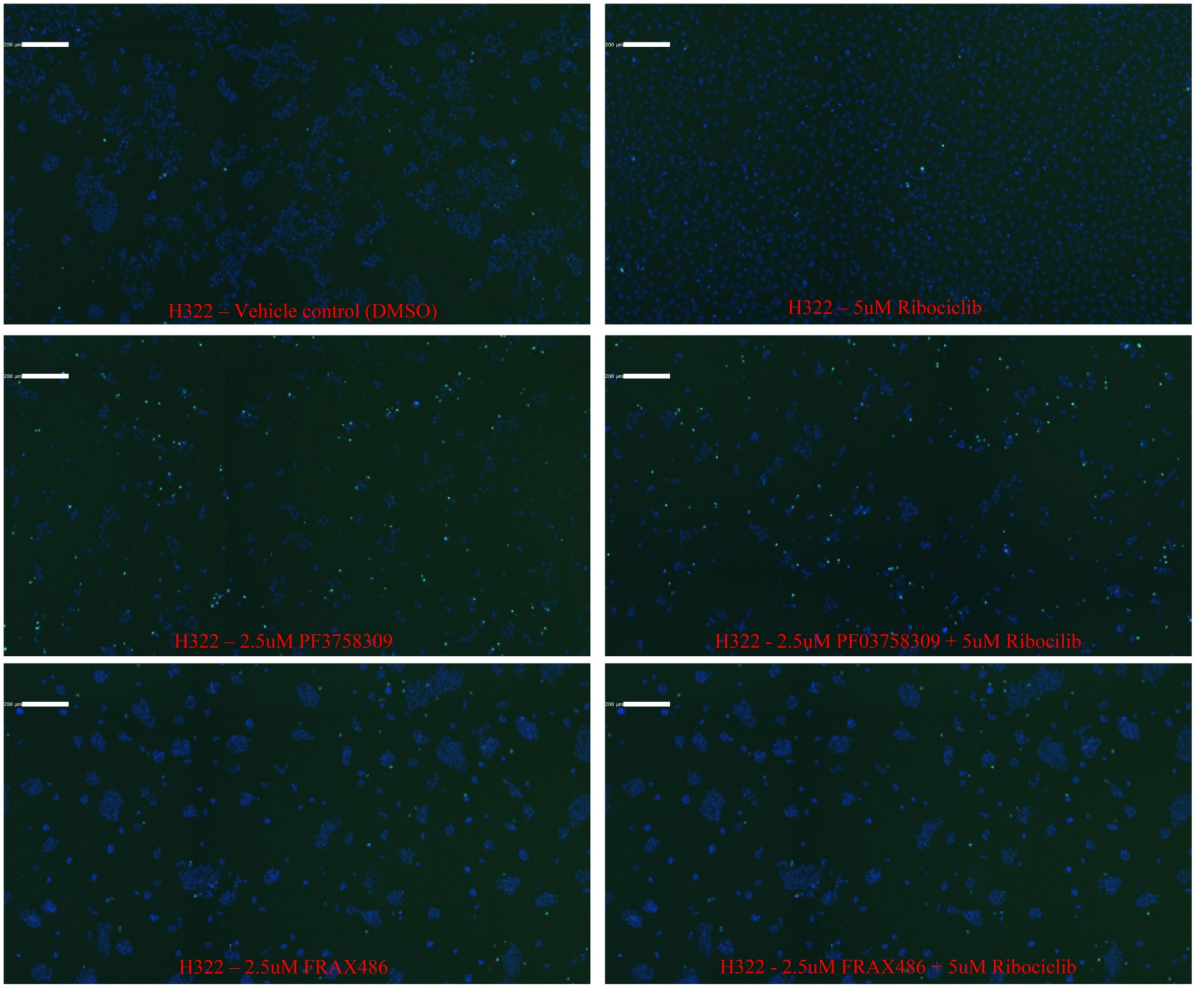
PF03758309 and its combinations induces apoptosis based on Celigo imaging. Colorimetric assay on a Celigo S (Nexcelom) showing apoptosis in H322 cells treated with vehicle control (DMSO), 2.5 μM PF03758309, 2.5 μM FRAX486 or 5 μM Ribociclib as well, as the combinations. The cells were stained with Cyto-ID green, to detect Caspase 3/7 (green), and Hoechst, to detect the nuclei of the cells (blue), and images were captured with the Celigo S.

PARP and caspase 3 cleavage assays were next performed to determine if apoptosis contributes to reduced viability measured by CTG assay. We examined apoptosis under the same conditions used for the cell cycle experiments. We tested PARP and caspase3 cleavage by immunoblot as well as by a colorimetric assay on a Celigo S (Nexcelom). The immunoblot (Fig 3) shows that in H322 PF03758309 alone partially induces apoptosis at 48 hours, but stronger in combination with Ribociclib. FRAX486 alone has a very weak apoptotic effect and none is detected for Ribociclib alone or in combination with FRAX486. However, a very high FRAX486 concentration (10 μM) results in apoptosis as well, as seen for PF03758309. Using the Celigo imaging cytometer, apoptosis was quantified at 48 hours using a caspase3/7 dye normalized to Hoechst staining (Table 3). In H322 (Fig 4) cells apoptosis increased from 15.7% for PF03758309 alone to 27.7% in combination with Ribociclib, while apoptosis with FRAX486 treatment was low and did not change between drug alone or in combination (3.12% and 3.37% respectively). For H157 cells (S6 Fig) a similar trend was observed, but overall apoptosis was significantly lower (PF03758309 alone 3.7% and in combination with Ribociclib 6.0%).

**Table 3:** Apoptosis in H322 cells or H157 cells.

Cell Line	DMSO	PF375	FRAX486	Ribo	PF375 + Ribo	FRAX + Ribo	high FRAX486	high FRAX + Ribo
H322	1.34 ± 0.07^a^	15.67 ± 1.75^a^	3.12 ± 0.73^a^	1.91 ± 0.84^a^	27.71 ± 2.05^a^	3.37 ± 0.17^a^	16.36	19.95
H157	0.36	3.69	0.53	0.31	6.04	0.48	nt	nt

Apoptosis in H322 cells or H157 cells (apoptosis as fraction of total cell ± standard error) after drug exposure for 48 hours, measured by fluorescent detection of caspase3/7 apoptosis and total cell detection by Hoechst on Celigo S. High FRAX486 = 10 μM, Abbreviation: PF375 = PF03758309, nt = not tested

^a^ n = 4, all others n = 1.

### Synergy and apoptosis are marked by the absence of autophagy—Autophagy is marked by the absence of synergy and apoptosis

**Fig 5:**
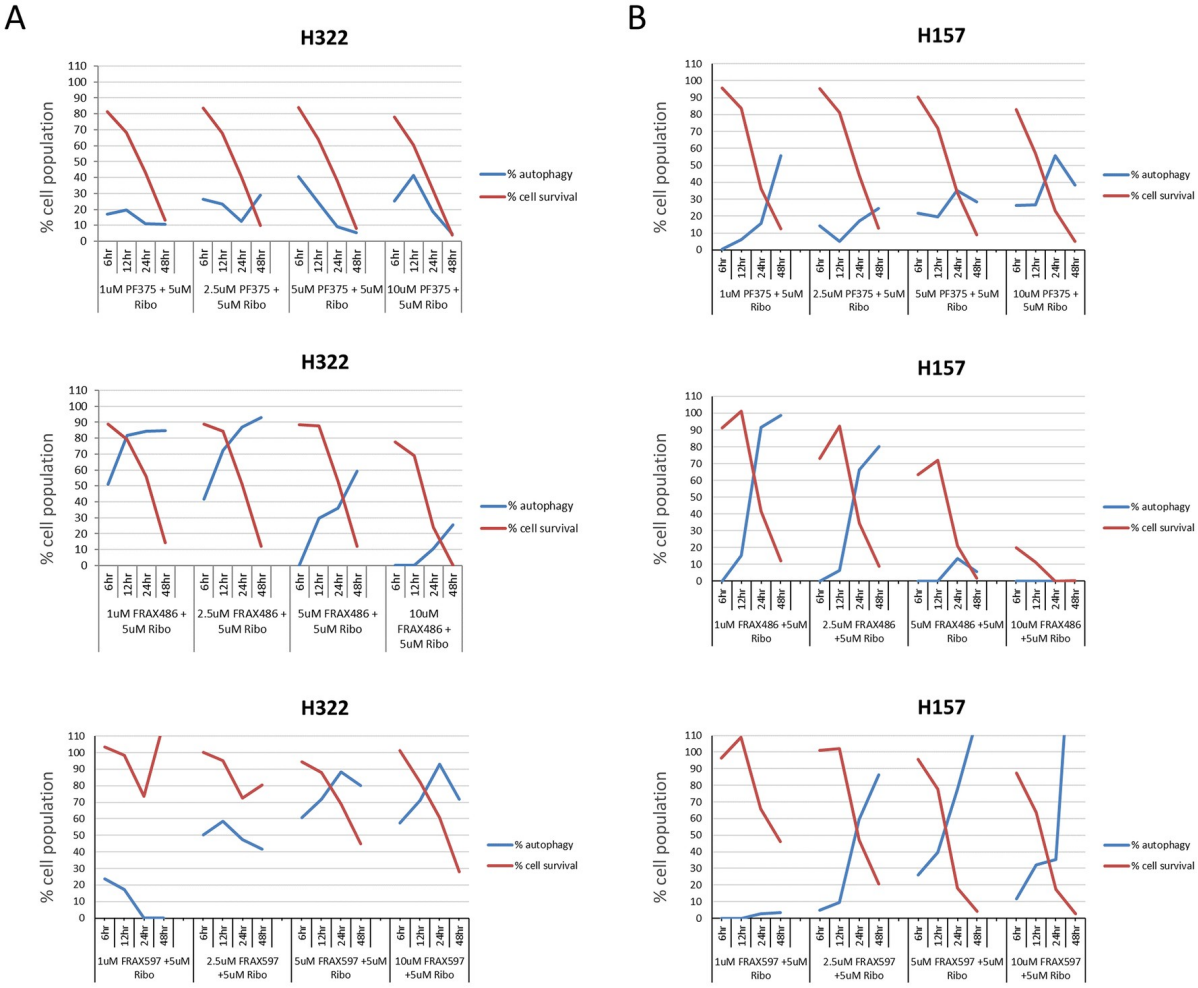
Autophagy and cell survival. Cells were treated with increasing concentrations of PF03758309, FRAX486 or FRAX597 in combination with 5 μM Ribociclib. After 6, 12, 24 and 48 hours, the cells were stained with Hoechst and Cyto-ID green to detect the cellular nucleus and autophagosomes, respectively. Cell survival was reported as a percentage of viable cells compared to the vehicle control, and autophagy was reported as number positive vesicles per viable cell normalized to vehicle control. Red lines % cell survival, blue lines % autophagy. Results shown in **A** and **B** are representative of H322 (n = 3) and H157 (n = 1) cell lines, respectively. The very high calculated percentage of cells in autophagy observed in H157 cells at high drug concentrations and later time points results from the fact that there are very few surviving cells under the conditions, and they all have autophagy.

Treatment of H322 and H157 cells with FRAX486 alone or in combination with Ribociclib does not result in apoptosis at the drug levels examined but does occur when FRAX486 concentrations are increased. This prompted us to examine the cells treated with the various drug combinations for autophagy under similar conditions as done for apoptosis (no consecutive drug addition). The presence of autophagosomes is an indicator of autophagy. Therefore, we measured if we could detect the appearance of autophagosomes on the Celigo, using the Cyto-ID autophagy kit. To get an accurate cell count and analyze the cell survival at the same time as autophagy, the cells were also stained with Hoechst 33342. H322 cells were treated with single PF03758309, FRAX486, FRAX597 or Ribociclib at increasing concentrations while H157 cells were only treated here with single FRAX597 (S7 Fig). Both lines were treated with PAKi at increasing concentration combined with 5 μM Ribociclib (Fig 5). Treatment with PF03758309 shows relatively low levels of autophagy compared to FRAX486 and FRAX597 which show much higher levels of autophagy. When combining drugs, the autophagy and survival patterns are similar to the PAKi alone. PF03758309 + Ribociclib overall has low staining for autophagy while Ribociclib combined with FRAX486 or FRAX597 has high autophagy. There is one exception to this pattern at high concentrations of FRAX486. The combined data for the autophagy and survival for all time points and drug concentrations clearly shows this pattern (Fig 5). When using high concentrations of FRAX486 (starting at 5 μM, but more clearly evident at 10 μM) autophagy is lower at all time points when compared to the lower FRAX486 concentrations, which does not occur with FRAX597.

In addition to the striking difference in staining for autophagosomes, the visual cell distribution pattern at lower concentrations of FRAX486, is distinctly different from the pattern for PF03758309 as shown for H322 (S8 Fig). But at high concentration of FRAX486, the cell distribution pattern starts to resemble the pattern seen for PF03758309. Ribociclib treatment, at 5 μM, as used in all drug combinations, shows almost no autophagy although autophagy does occur at 20 μM (not used in combination with PAKis).

### Inhibition of PAK1-4 is necessary for the synergy between PF03758309 and Ribociclib

The data in Table 4 demonstrate that high concentrations of FRAX486 (10 μM) mimic the apoptosis induced by PF03758309. To confirm that inhibition of PAK4 is responsible for this difference, we treated H322 cells for 48 hours with siRNA to PAK4, followed by 24 hours of FRAX486 with and without Ribociclib (S9A Fig). PAK4 knockdown should eliminate any scaffolding activity in addition to blocking its kinase activity while FRAX486 will inhibit the kinase activity of PAK1-3. Surprisingly, PAK4 knock down results in upregulation of both PAK1 and PAK2 (S9B Fig). For this reason, PAK4 knock down may not result in an overall reduction in PAK activity, making it more difficult to show the apoptotic effect. Based on caspase 3 cleavage, knock down of PAK4 alone or combined with Ribociclib does not result in apoptosis. However, knock down of PAK4, combined with inhibition of PAK1-3 via FRAX486, with or without Ribociclib, results in cleaved caspase 3. A result that suggest PAK4 inhibition is the key difference between FRAX486 and PF03758309. Since PAK5 is not expressed in our model cell line H322 and PAK6 is down regulated by Ribociclib, we conclude that inhibition of PAK1-4 is most likely the reason for synergy between PF03758309 and Ribociclib.

**Table 4:** Summary of drug treatment phenotype.

PAKi			CDK4/6i	Phenotype		
PF03758309	FRAX486	FRAX597	Ribociclib	cell cycle arrest	apoptosis	autophagy
+	-	-	+	G1/Go and G2	+++	+/-
-	+	-	+	G1/Go	+/-	+++
-	+++	-	+	nt^a^	+++	+/-
-	-	+	+	G1/Go	-	+++

The left side of the table shows the treatment (“-”indicates no drug, “+” indicates standard drug concentrations and “+++” indicates high drug concentrations, as defined in the text). The right side of the table shows a relative quantitation for the three type of observations: Cell cycle arrest, apoptosis, autophagy. nt^a^ = not tested.

## Discussion

Lack of cell cycle control is a hallmark of cancer. The CDK/Rb/E2F pathway is essential for the controlled execution of the cell cycle. CDK4/6is, Palbociclib, Ribociclib and Abemaciclib, have been effectively used in combination therapy [34,35], and have shown to be synergistic with drugs targeting other oncogenic pathways, including MEK, KRAS, BRAF, and ALK, in various cancers [4,13,14,36–39]. To find additional pathways and targets, we performed a drug combination screen for synergy. The results of the screen, revealing the pan-PAK inhibitor PF03758309 to be synergistic with CDK4/6is, were validated, including Ribociclib as a third CDK4/6i. Another PAKi, FRAX486, although present in the library, did not show synergy and this result was validated as well. The results show conclusively that PF03758309 is synergistic with all three of the CDK4/6is tested, while FRAX486 is not and at some concentrations may even be antagonistic. The main difference between the two inhibitors is that PF03758309 is a pan-PAK inhibitor (all 6 PAKs), while FRAX486 only inhibits PAK1-3 combined with a much weaker inhibition of PAK4 (IC_50_—PF03758309: 1.3nM, FRAX486: 575/779nM). Interestingly, looking at the synergy matrices, it is evident that, although overall antagonistic, a slightly synergistic response can be seen at higher concentration of FRAX486 where PAK4 is inhibited as well, consistent with the drug behaving like PF03758309. Another PAKi that exclusively inhibits PAK1-3, FRAX597 was added to show that without PAK4 inhibition, no synergy occurs.

Examination of the cell cycle showed PF03758309 to result in a G1/G0 as well as a G2M arrest while FRAX486, FRAX597 and Ribociclib results only in a G1/G0 arrest. Experimental results further revealed that apoptosis occurs for PF03758309 alone, as well as in combination with Ribociclib. By itself or in combination, FRAX486 and Ribociclib do not cause apoptosis, with one exception: when FRAX486 concentrations are increased to 10 μM, apoptosis is evident by activation of caspase3/7. Before this observation, off-targets of PF03758309 were still on the list of possibilities [32], but increasing FRAX486 concentrations can increase the inhibition of PAK4, that otherwise is only weakly inhibited by FRAX486. Including PAK4 in the target range appears to be responsible for the drug responding more like PF03758309. Exploring the possibility that autophagy plays a role, possibly preventing apoptosis, cells treated with PF03758309 have low levels of autophagy, while cells treated with FRAX486 and FRAX597 show high levels of autophagy. Again, at higher concentrations FRAX486 starts to behave like PF03758309. This is true both alone and in combination with Ribociclib. However, this effect does not occur with FRAX597 since there is no inhibition to PAK4. To our knowledge, the role of the PAK family in the autophagy versus apoptosis decision has not previously been observed, nor has this information been incorporated in the rationale for drug development.

PAKs were discovered as binding proteins to small GTPases [40]. PAK1, 2, 4 and 5 have all been shown to have the ability to protect cells from apoptosis. PAK1, 4 and 5 directly phosphorylate and thereby inactivate pro-apoptotic BAD [41]. PAK1, 4 and 5 (by different mechanisms) support activation of AKT via the PI3K pathway and PAK1 can directly be activated by AKT. AKT can also directly phosphorylate BAD. Phosphorylation of BAD inhibits its dimerization with the anti-apoptotic factors Bcl-xl and Bcl2. These in turn can prevent the activation of effector caspases such as caspase3. In addition, PAK4 inhibits the initiator caspase 8 independent of kinase activity. Full length PAK2 has anti-apoptotic activity, but under certain stress conditions it is cleaved and a pro-apoptotic fragment assists in the apoptotic response [17,20,24,41–43]. In addition to involvement in protection from apoptosis, PAK1 and 4 are involved in cell survival via the RAS/PI3K/AKT as well as the MAPK pathway [44,45]. From all these reports it has become increasingly clear that PAKs have overlapping functions [17]. Some of our experiments done with RNAi also indicate that removing one PAK can increase the presence of other PAKs. We conclude therefore that the differences seen in our data between the pan PAK inhibitor PF03758309 and the (primarily) group I PAK inhibitor FRAX486 as well as FRAX597 is the result of one or more of the group II PAKs compensating for the loss of activity of one or more of the group I PAKs. Our results strongly point to PAK4 as the required group II PAK, while no information is yet available on the group I PAKs. Using RNAi to PAK4, we tested the hypothesis that of the group II PAKs, inhibition of PAK4 is needed to achieve apoptosis when using FRAX486 or FRAX486/Ribociclib. Results for this experiment were weak, since knock down of PAK4 resulted in the upregulation of PAK1 and PAK2. Nevertheless, we showed that knock down of PAK4 combined with FRAX486 (PAK1-3 inhibition), with or without Ribociclib, results in apoptosis. Knock down of PAK4 alone or combined with Ribociclib does not. This, combined with the observation that PAK5 is not expressed in our model cell line H322 and PAK6 (low expression) is down regulated by Ribociclib, allowed us to surmise that inhibition of a combination of PAKs is the most likely reason for synergy. Although off-targets are known for PF03758309, the research presented here shows the effect of the drug is due to the combined inhibition of the PAKs. The research presented here indicates that drug synergy may well depend on specific combinations of PAK inhibition. While PAK inhibitors, so far, have gained little traction in the clinic due to off target effects on healthy cells, one of the excellent reviewers of this manuscript pointed out that recent work [46] has demonstrated that targeting of PKCiota-PAK1 signaling pathways is highly effective in preclinical models of NSCLC. In this context, our work may have clinical relevance.

In summary, herein we find that the pan-PAK inhibitor, PF03758309, acts synergistically with multiple CDK4/6i in multiple NSCLC cell lines. Lack of synergy by another PAK inhibitor, FRAX486, led us to discover that group I PAKs play a major role in the regulation of autophagy and a combination of the six-member PAK family regulates the decision to undergo apoptosis or survive by the induction of autophagy upon inhibition of CDK4/6. We believe selective inhibition of the unique PAK combination responsible for synergy could greatly improve the efficacy of CDK4/6 inhibition and broaden the spectrum of cancer treatment.

## Experimental procedures

### Combination drug screen

Drug combination screening was done in 4 NSCLC cell lines (H157, H322, H1299 and H2170, see S1 Table) with a customized library of 240 targeted agents and either palbociclib or abemaciclib as base drugs. Cells were plated at 500–1000 cells/well in black, clear bottom 384 well plates. After 24 hours, drugs were added to the cells. Cell viability was determined in duplicate using the CellTiter-Glo (CTG) assay after 72 hours of drug exposure with 0.5 and 2.5 μM of library compounds in the presence of DMSO, 2.5 μM palbociclib or 0.75 μM abemaciclib. The viability ratio was determined by dividing the average viability of DMSO-treated cells with the average viability of CDK4/6 inhibitor-treated cells. Screening results are shown in S2 Table.

### Cell culture

Cell lines, H157, H322, H1299, H2170, A427, HCC4006, H1648, HCC827, H1437, H1944, H2172 and HBEC were provided by the Moffitt Lung Cancer Center of Excellence Cell Line Core after testing negative for mycoplasma and authentication by short tandem repeat (STR) analysis. Cells were cultivated in RPMI-1640 media with 10% FBS (complete RPMI), except for HBEC, which was grown in Keratinocyte Serum Free Medium w/EGF and BPE, and maintained in the incubator with 5% CO2 at 37°C

### Synergy validation with viability assays

For validation of drug synergy between Ribociclib and PF03758309 and Ribociclib and FRAX486 in H157, H322, H1299 and H2170, cells were plated with a density of 250 (H1299), 500 (H157) or 1,000 (H322 and H2170) cells per well in a black, clear bottom 384 well tissue culture plate. After adhering for 24 hours, cells were treated with DMSO (VC), single drug or a drug combination and incubated for an additional 72 hours. All treatments were done in duplicate. Inhibitors were diluted in complete medium to 10x the final concentration and added to the wells at 1/10 final well volume. For PAKi the dilution range used was five concentrations in 4-fold serial dilutions starting with the highest final concentration of 10 μM. For the CDK4/6i the five concentrations at 4-fold serial dilutions started at 20 μM. Cell viability was measured with CellTiter-Glo Luminescent Cell Viability Assay reagent (Promega). After addition of CellTiter-Glo reagent, plates were shaken in the dark at 400 rpm for 5 minutes and placed in the dark for another 10 minutes without shaking. An M5 Spectramax plate reader (Molecular Devices) using 100 ms integration was used to collect the data. Data were analyzed using the Bliss model of independence.

### Cell cycle analysis

H322, H157 and H1299 cells were plated at a density of 1.5, 0.8 or 0.4x10^6^ per P100, respectively. After 24 hours cells were treated with DMSO, single drug or combination of drugs (as indicated in the figures) for 24 hours. If treated consecutively, one drug was applied at first time of addition. The second drug was applied 6 hours later for an 18 hours treatment. To harvest, medium was collected, and cells were trypsinized and combined with the medium. Cells were counted, washed with PBS and after straining, fixed with 70% ethanol and stored at -20°C overnight. The next day cells were washed with PBS resuspended in PBS/0.25% Triton X-100 at 10^6^cells/mL and incubated for 15 min on ice. The permeabilized cells were washed with PBS, resuspended in PBS/1%BSA +1:400 α-phospho Ser10 histone H3 (pHH3)(Cell Signaling #3377) and incubated for 2 hours at room temperature. After washing twice with PBS/1%BSA, 100 μL PBS/1%BSA with 0.5 μL goat anti Rabbit-Alexa Fluor 647 (Molecular Probes A21244 or A31573) was added and cells were incubated for 1 hour at room temperature in the dark. Cells were washed twice with 3mL PBS and resuspended in a final volume of 0.5mL PBS with 1μg/mL (final) DAPI. Cells were incubated at 4°C (in the dark) overnight. Cells were acquired using a FACSCanto II with DIVA 8.01. The cell cycle analysis was done with ModFit LT 3.2.1. The pH3 analysis was done with Flowjo version 10.

### Cell lysate preparation for immunoblotting

Cells were harvested by scraping and collected in PBS, pelleted by centrifugation, and lysed with RIPA lysis buffer (Fisher AKR 191) containing proteinase and phosphatase inhibitors (Sigma, PPC1010-1ML) for 20 min with frequent agitation. Samples were centrifuged maximum speed in a micro centrifuge at 4°C for 20 min and supernatant was collected. Protein concentration was determined using a Bradford assay (Bio-Rad, 500–0006).

### Immunoblotting

Equal amounts of total cell lysates were separated by SDS-PAGE and transferred to activated PVDF membranes using a wet transfer system (Bio-Rad). Membranes were blocked and probed with primary and secondary antibodies according to standard techniques. Images were acquired using an Odyssey FC Imager (LI-COR). Immunoblotting was performed with primary antibodies against actin (Sigma cat# SAB1305567), PARP1 (Cell Signaling, #9542), cleaved caspase-3 (Asp175) (Cell Signaling, #9661), PAK4 (Cell Signaling, #3242), PAK1 (Cell Signaling, #2602), PAK2 (Cell Signaling, #2608), phospho Ser112-BAD (Cell Signaling, #5284), BAD (Cell Signaling, #9292).

### Apoptosis on Celigo Imaging Cytometer

Cells were plated with a density of 2000 (H157) or 4000 (H322) cells per well in a black, clear bottom 96 well tissue culture plate. After 24 hours incubation, medium was replaced with 100 μL/well of complete medium containing the desired drug mix (0.4% DMSO, 2.5 μM PF03758309 or FRAX486, 5 μM Ribociclib and combinations) and 2 μM Viastain live Caspase 3/7 reagent (Nexcelom and CS1-V0002-1). After an additional 48 hours incubation Hoechst 33342 dye (Fisher Scientific and H3570) was added at a final concentration of 4 μg/mL and incubated for 30 min more in the dark. Cells and apoptosis were visualized and quantified on a Celigo Imaging Cytometer using fluorescent imaging (Nexelcom).

### Autophagy on Celigo Imaging Cytometer

Cells were plated in duplicate at a density, respectively, for H322 and H157 of 2x10^4^ and 1.2x10^4^ (for 6 and 12 hours), 1x10^4^ and 0.6x10^4^ (24 hours) or 0.5x10^4^ and 0.3x10^4^ (48 hours) cells per well in a black, clear bottom 96 well tissue culture plate. After 24 hours incubation, medium was replaced with 100 μL/well of complete medium containing the desired drug mix (0.2% DMSO, 1, 2.5, 5 or 10 μM PF03758309, FRAX486 or FRAX597 2.5, 5, 10 and 20 μM Ribociclib or combinations of all PAKi concentrations with 5 μM Ribociclib. After 6, 12, 24 and 48-hour incubations, cells were stained with Cyto ID green (Enzo and ENZ-51031-K200) and Hoechst 33342 dye following the Enzo recommended protocol. In brief, the medium was aspirated, and cells washed with 100 μL 1x assay buffer/5%FBS. The wash buffer was replaced with 100 μL 1x assay buffer/5%FBS containing Cyto ID-green and Hoechst 33342 (both at 1:1000 dilution of stocks). Cells were incubated for 30 min 37°C protected from light, then washed 2x with 200 μL 1x assay buffer/5%FBS. The last wash was replaced with 100 μL 1x assay buffer/5%FBS. Stained cells were visualized and quantified on a Celigo Imaging Cytometer using fluorescent imaging (Nexelcom).

### RNA interference

siRNA SMART pools (Dharmacon) were re-suspended to make a 20 μM stock in 1x siRNA buffer (1:5 Thermo Scientific 5x siRNA Buffer (B-002000-UB-100) diluted with RNase-free water (B-002000-WB-100)) and mixed for 15 minute at room temperature. siRNA stocks were diluted to the desired final concentration in OptiMEM media (Fisher 31-985-062) and incubated for 5 minutes. siRNA was incubated for additional 20 minutes in well (to be used for plating cells) with lipofectamine mix (OptiMEM media plus lipofectamine RNAiMax (Fisher 13-778-150)) at a 1:1 ratio. Cells were plated at 2.5x10^5^ cell per well, incubated for 48 hours and treated with FRAX486 (2.5 μM), Ribociclib (5 μM) or the combination of drugs. Cells were incubated an additional 24 or 48 hours and harvested for analysis on immunoblot. In addition to siRNA, cells were treated with shRNA to PAK4. Two clones were obtained from Sigma, TRCN0000272579 and TRCN0000272510 (herein referred to as shPAK4(79) or shPAK4(10)). H322 was transduced with shNT, shPAK4(79), shPAK4(10) as well as with the combination of shPAK4(79) and (10). PAK4 knock down was examined by immunoblot.

## Supporting information

S1 Fig Synergy assays for PF03758309 and Ribociclib in multiple cancer cell lines. **A**. Synergy matrix (using the Bliss model of independence) for PF03758309 with Ribociclib for H157, H1299 and H2170. Image representative of n = 3. **B)** Sum of synergy for additional cell lines tested. All cell lines are Rb competent, except H2172, an Rb null cell line.
(TIF)

S2 Fig Synergy assays for FRAX486 and Ribociclib in multiple cancer cell lines. Synergy matrix (using the Bliss model of independence) for FRAX486 with Ribociclib for H157, H1299 and H2170. Image representative of n = 3.
(TIF)

S3 Fig Synergy assays for FRAX597 and Ribociclib in multiple cancer cell lines. Synergy matrix (using the Bliss model of independence) for FRAX597 with Ribociclib for H157, H322, H1299 and H2170. Image representative of n = 3.
(TIF)

S4 Fig EC_50_ values for each single drug used. Values were assessed by isolating the individual readout values for each drug from the synergy matrix and calculating the EC50 in GraphPad.
(TIF)

S5 Fig BAD phosphorylation at Serine 112 is reduced following treatment with PAKi. **A**. An immunoblot using antibodies to BAD phosphorylation at Serine 112 was used as an in vivo marker for PAK enzymatic activity. B. Immunoblot data was quantitated and the phosphserine112 BAD signal plotted as a percentage of the total BAD signal.
(TIF)

S6 Fig Apoptosis assays based on Celigo imaging of caspase 3/7 cleavage. H157 cells were treated with vehicle control (DMSO), 2.5 μM PF03758309, 2.5 μM FRAX486 and 5 μM Ribociclib or combinations, as indicated. The cells were stained with Cyto-ID green to detect Caspase 3/7 cleavage (green) and Hoechst to detect the nuclei (blue).
(TIF)

S7 Fig Autophagy and cell survival. Cells were treated with increasing concentrations of PF03758309, FRAX486, FRAX597 or Ribociclib. After 6, 12, 24 and 48 hours, the cells were stained with Hoechst and Cyto-ID green to detect the cellular nucleus and autophagosomes, respectively. Cell survival was reported as a percentage of viable cells compared to the vehicle control, and autophagy was reported as number positive vesicles per viable cell normalized to vehicle control. Red lines % cell survival, blue lines % autophagy. Results shown representative of n = 3 for H322 (all 4 drugs) and n = 1 for H157 (FRAX597 only. For H157 the other PAKis were only done in combo with Ribociclib).
(TIF)

S8 Fig Autophagy and cell survival. Cells were treated as described for Figs 4 and S8. The presence of live cells and autophagosomes visualized by fluorescence using the Celigo is shown here at 24 hours for H322. Blue is Hoechst stain, Green is Cyto-ID stain. All images show an ~500 μM slice of the total well image.
(TIF)

S9 Fig PAK inhibition and apoptosis. **A**. H322 cells treated with siNT (non-targeting) or siPAK4 for 72 hours (time needed for maximum PAK4 knock down) and FRAX486 and/or Ribociclib for the last 24 hours (treated at 48 hours). Apoptosis was measured by immunoblot detection of cleaved Caspase3 and PARP. **B**. Immunoblot showing the effect of PAK4 knock down on PAK1 and PAK2 protein expression, using H322 shNT, shPAK4(79) or shPAK4(10) alone, or a combination of shPAK4(79) and (10).
(TIF)

S1 Table Genotypes and key mutations in the cell lines used in this study. Key driver mutations and wild-type genes are highlighted. The information provided is based on data downloaded from the cancer cell line encyclopedia (https://portals.broadinstitute.org/ccle).
(XLSX)

S2 Table Combination screening results. Viability assays were performed in 4 NSCLC cell lines (H157, H332, H1299 and H2170) with a customized library of 240 targeted agents in combination with palbociclib or abemaciclib. The library drug chemical names, clinical synonyms and presumed primary drug targets are indicated. The viability ratio was determined by dividing the average viability of DMSO-treated cells with the average viability of CDK4/6 inhibitor-treated cells and depicted in a heat map. Combinations with potential synergy are highlighted in red and thus that may be antagonistic are highlighted with green.
(XLSX)

S1 Raw images This pdf annotates the uncropped original Western-Blot gel images for Figs 3, S5 and S9 captured by the LI-COR instrument and exported in tiff format. (PDF)

## Abbreviations

AKT: a serine/threonine protein kinase
ALK: Anaplastic lymphoma kinase
BAD: BCL2 associated agonist of cell death
Bcl2: B-cell lymphoma 2
Bcl-xl: B-cell lymphoma-extra large
BRAF: serine/threonine-protein kinase B-Raf
CDC42: Cell division control protein 42 homolog
CDK: Cycle Dependent Kinase
CTG: Cell Titer Glo
DAPI: 4′,6-diamidino-2-phenylindole
E2F: Eukaryotic Transcription Factor
EGFR: Epidermal growth factor receptor
GTPases: Guanosine Tri-phosphatases
KRAS: a GTPase
mTOR: mammalian target of rapamycin
NSCLC: Non-Small Cell lung cancer
PAK: P21 Activated Kinase
pHH3: α-phospho Ser10 histone H3
PI3K: Phosphoinositide 3-kinase
Rb: Retinoblastoma
