# Supplementary figures and images for: CDK4/6 inhibition synergizes with inhibition of P21-Activated Kinases (PAKs) in lung cancer cell lines
Source: PLoS One. 2021 Jun 17;16(6):e0252927. doi: 10.1371/journal.pone.0252927 (PMC8211232; doi:10.1371/journal.pone.0252927)

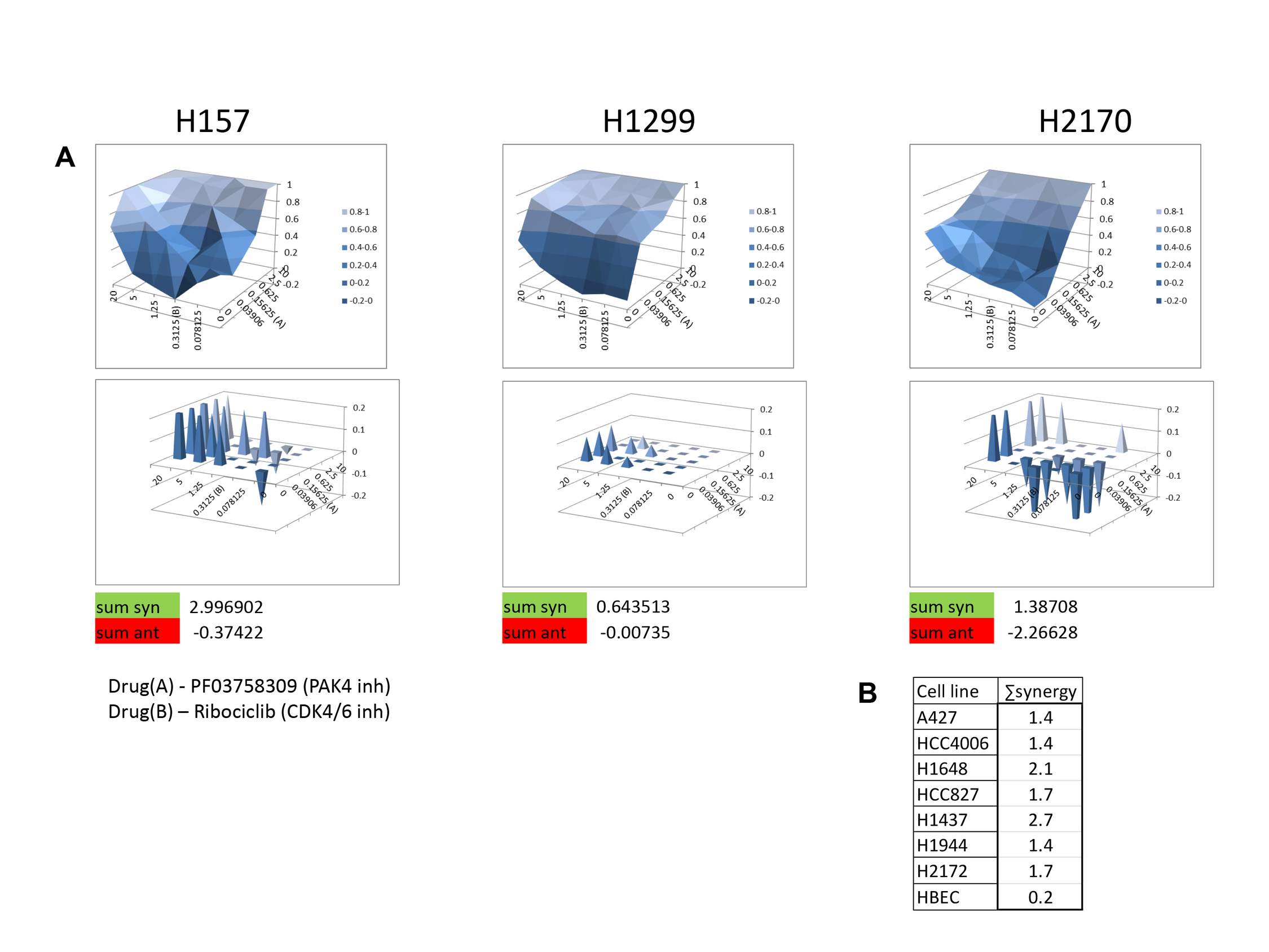

Supplement: S1 Fig — A. Synergy matrix (using the Bliss model of independence) for PF03758309 with Ribociclib for H157, H1299 and H2170. Image representative of n = 3. B) Sum of synergy for additional cell lines tested. All cell lines are Rb competent, except H2172, an Rb null cell line. (TIF) [file pone.0252927.s001.tif]

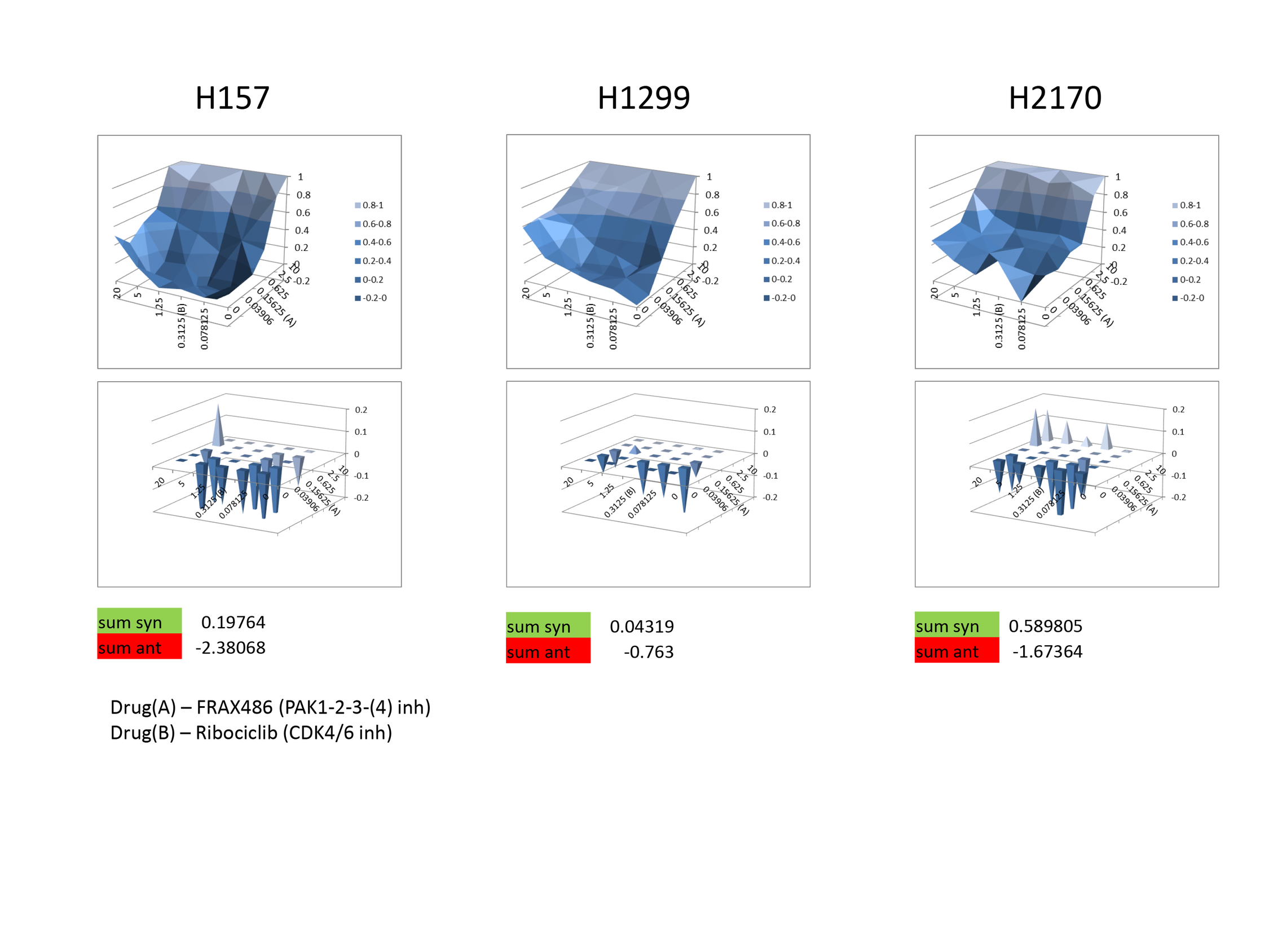

Supplement: S2 Fig — Synergy matrix (using the Bliss model of independence) for FRAX486 with Ribociclib for H157, H1299 and H2170. Image representative of n = 3. (TIF) [file pone.0252927.s002.tif]

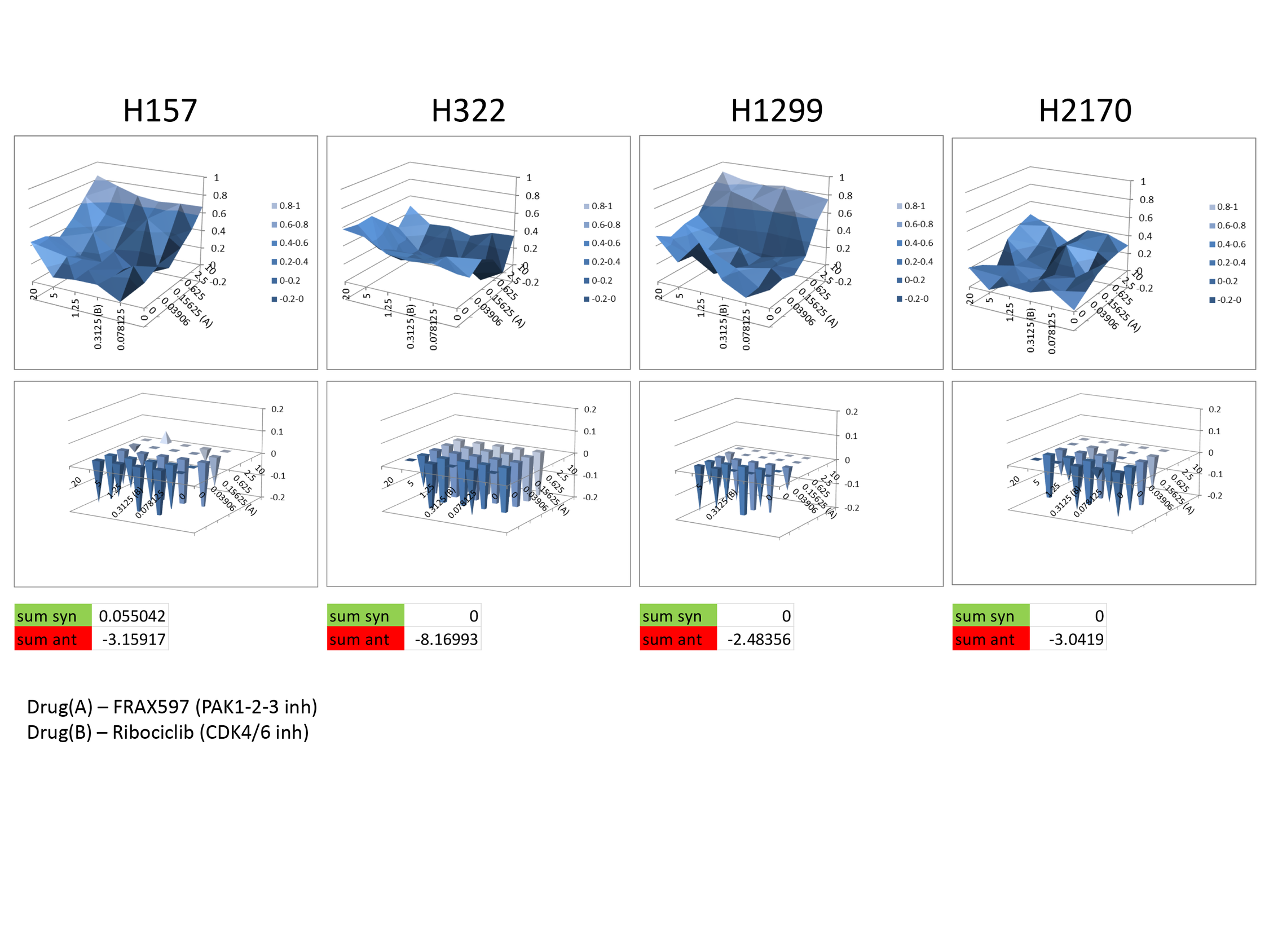

Supplement: S3 Fig — Synergy matrix (using the Bliss model of independence) for FRAX597 with Ribociclib for H157, H322, H1299 and H2170. Image representative of n = 3. (TIF) [file pone.0252927.s003.tif]

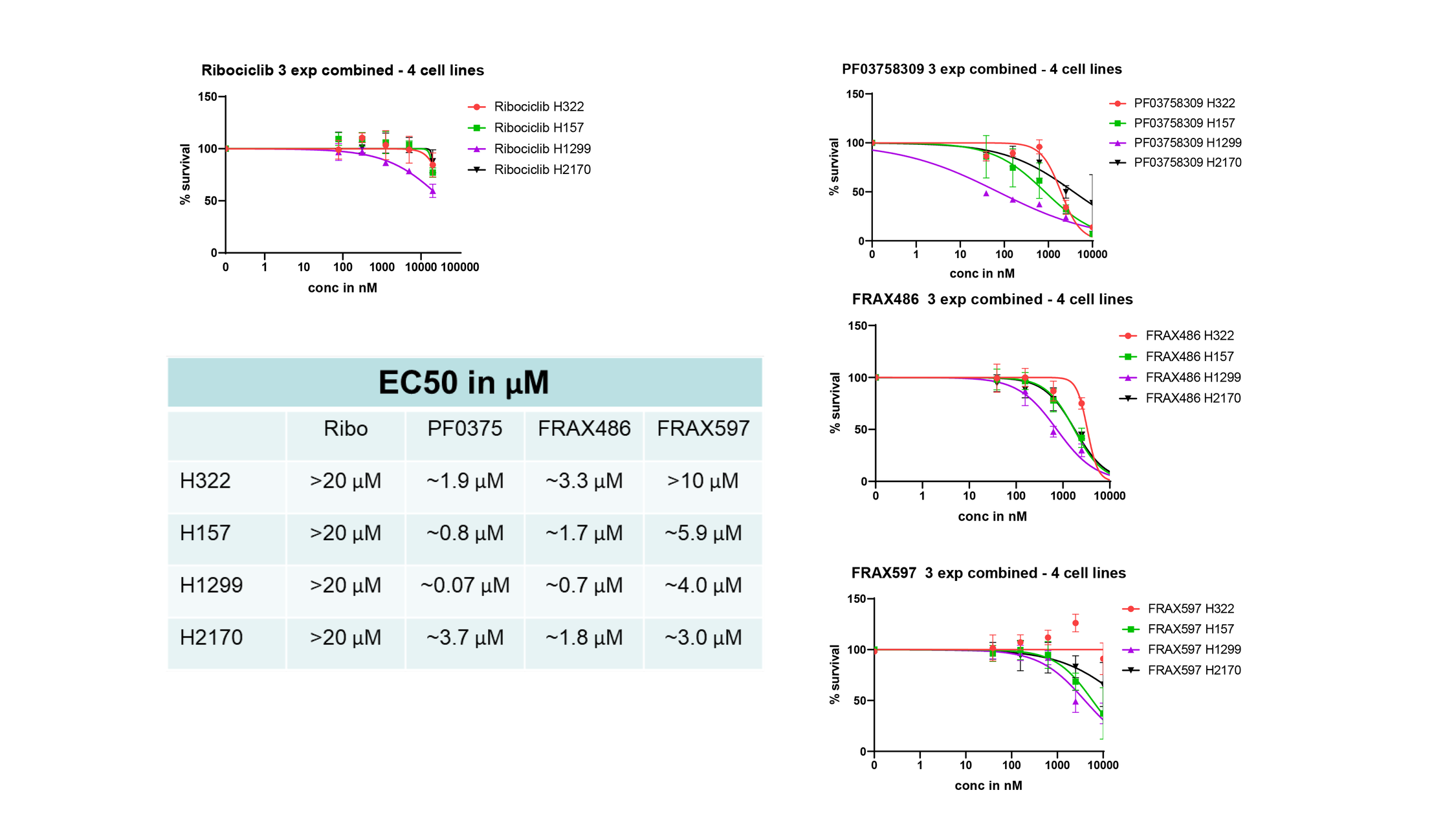

Supplement: S4 Fig — Values were assessed by isolating the individual readout values for each drug from the synergy matrix and calculating the EC50 in GraphPad. (TIF) [file pone.0252927.s004.tif]

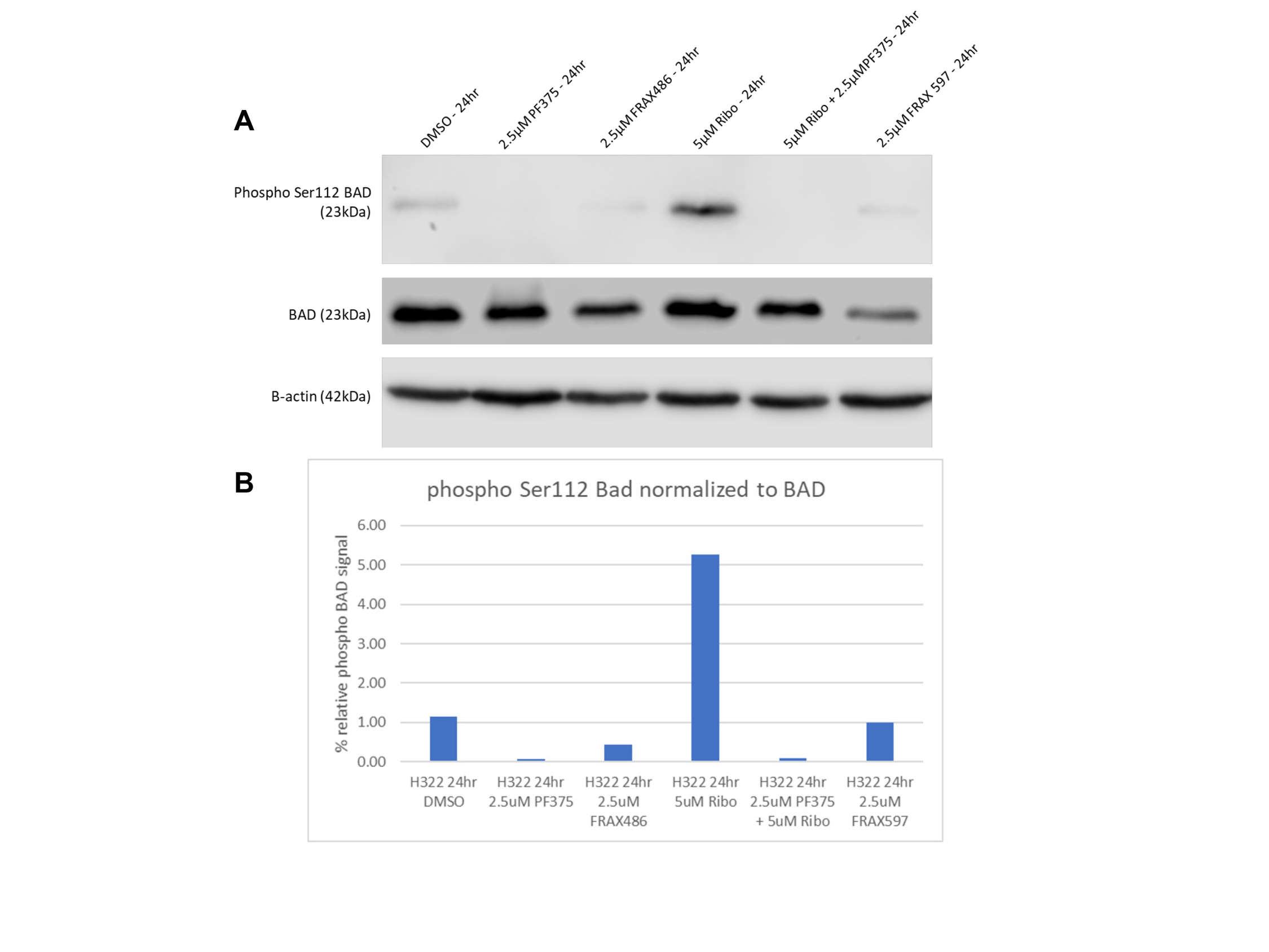

Supplement: S5 Fig — A. An immunoblot using antibodies to BAD phosphorylation at Serine 112 was used as an in vivo marker for PAK enzymatic activity. B. Immunoblot data was quantitated and the phosphserine112 BAD signal plotted as a percentage of the total BAD signal. (TIF) [file pone.0252927.s005.tif]

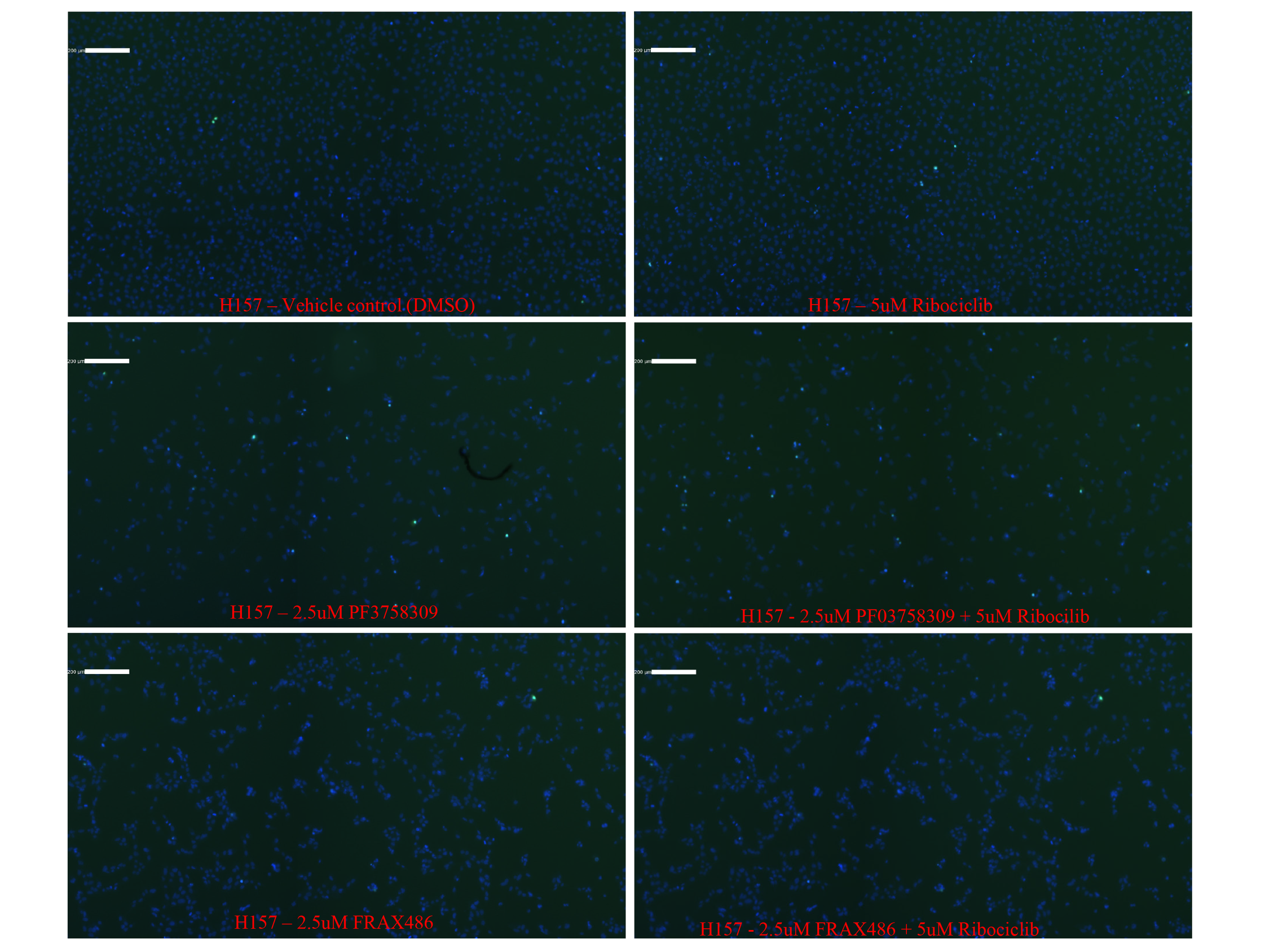

Supplement: S6 Fig — H157 cells were treated with vehicle control (DMSO), 2.5 μM PF03758309, 2.5 μM FRAX486 and 5 μM Ribociclib or combinations, as indicated. The cells were stained with Cyto-ID green to detect Caspase 3/7 cleavage (green) and Hoechst to detect the nuclei (blue). (TIF) [file pone.0252927.s006.tif]

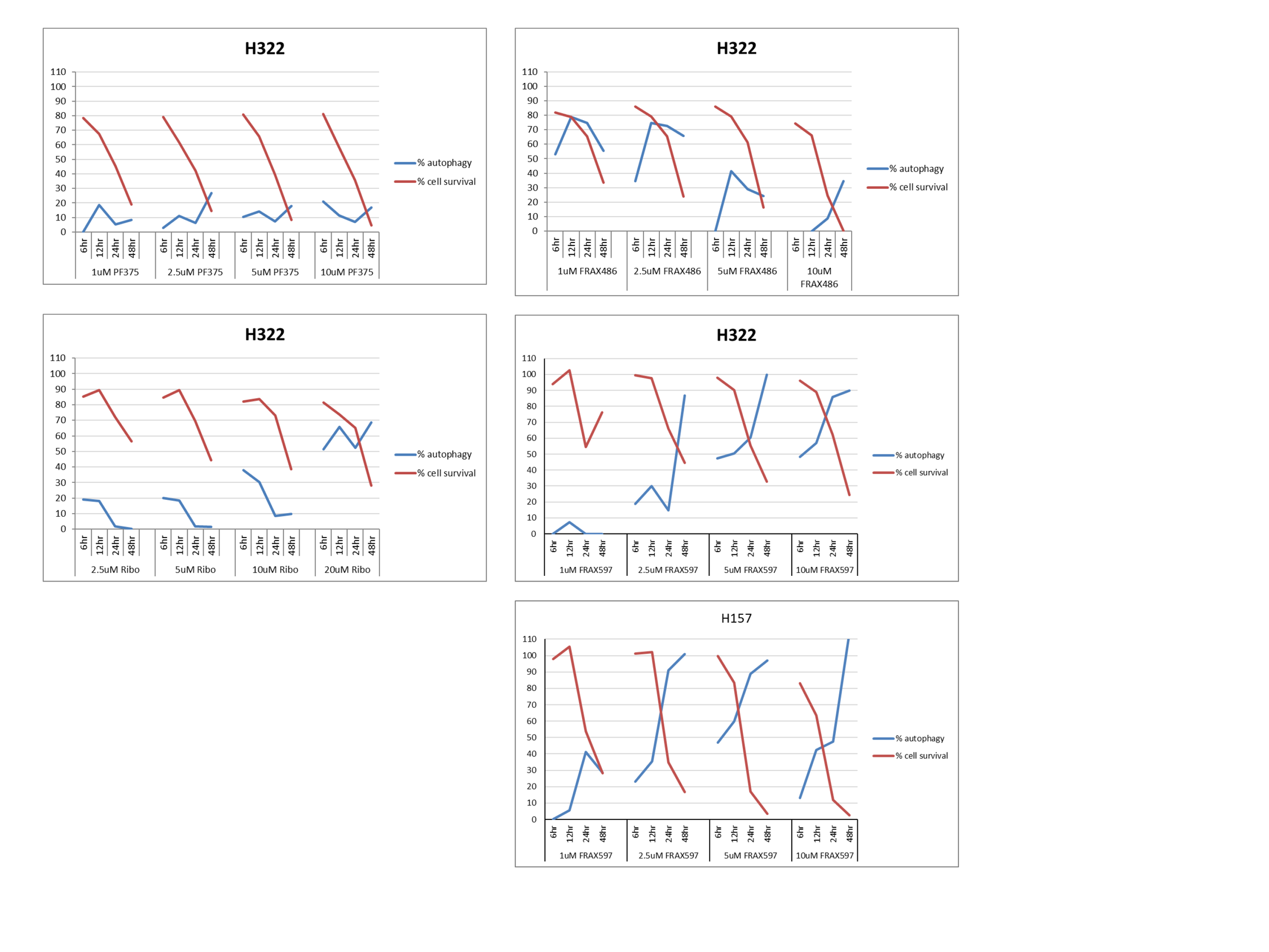

Supplement: S7 Fig — Cells were treated with increasing concentrations of PF03758309, FRAX486, FRAX597 or Ribociclib. After 6, 12, 24 and 48 hours, the cells were stained with Hoechst and Cyto-ID green to detect the cellular nucleus and autophagosomes, respectively. Cell survival was reported as a percentage of viable cells compared to the vehicle control, and autophagy was reported as number positive vesicles per viable cell normalized to vehicle control. Red lines % cell survival, blue lines % autophagy. Results shown representative of n = 3 for H322 (all 4 drugs) and n = 1 for H157 (FRAX597 only. For H157 the other PAKis were only done in combo with Ribociclib). (TIF) [file pone.0252927.s007.tif]

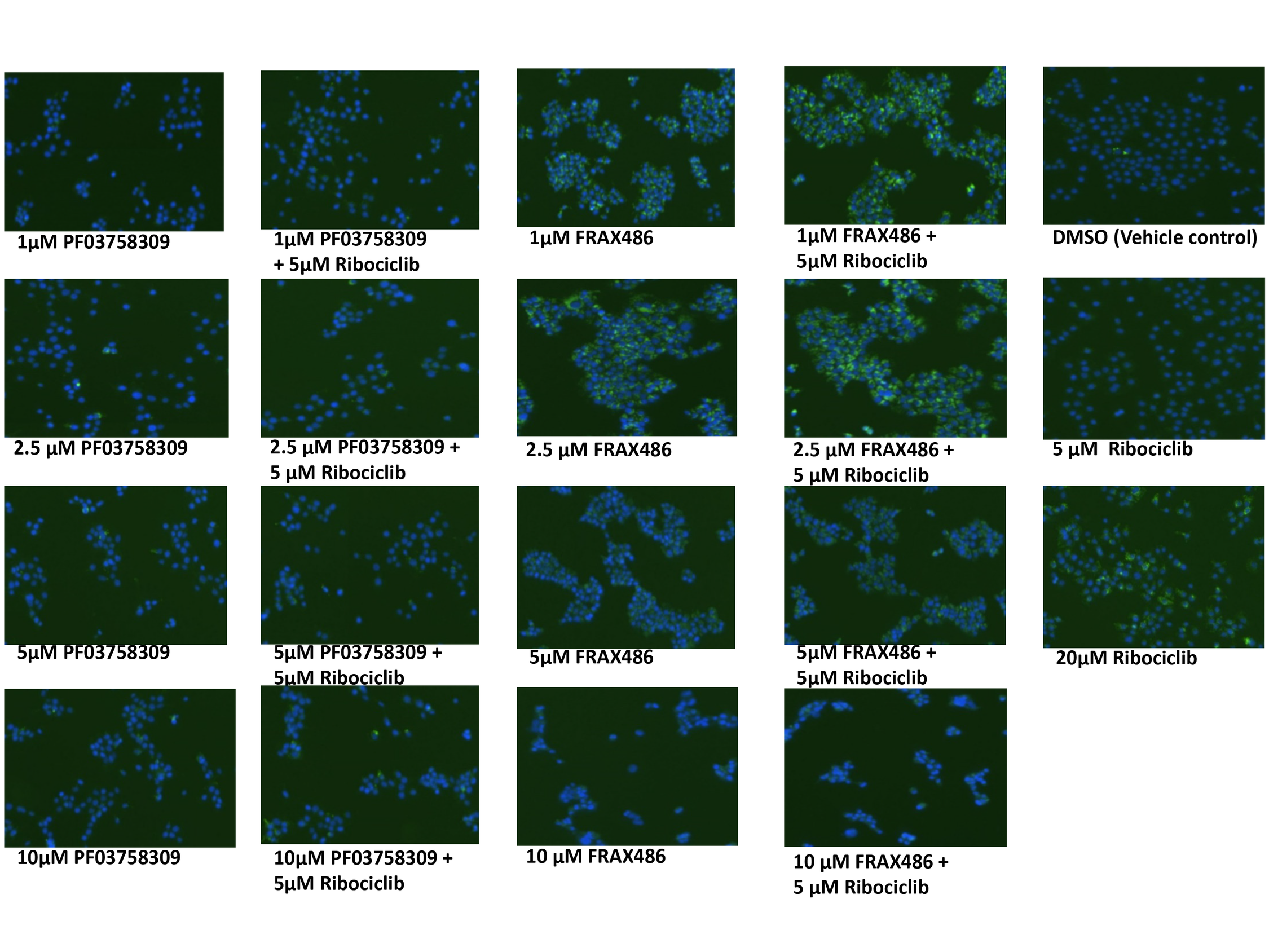

Supplement: S8 Fig — Cells were treated as described for Figs 4 and S8. The presence of live cells and autophagosomes visualized by fluorescence using the Celigo is shown here at 24 hours for H322. Blue is Hoechst stain, Green is Cyto-ID stain. All images show an ~500 μM slice of the total well image. (TIF) [file pone.0252927.s008.tif]

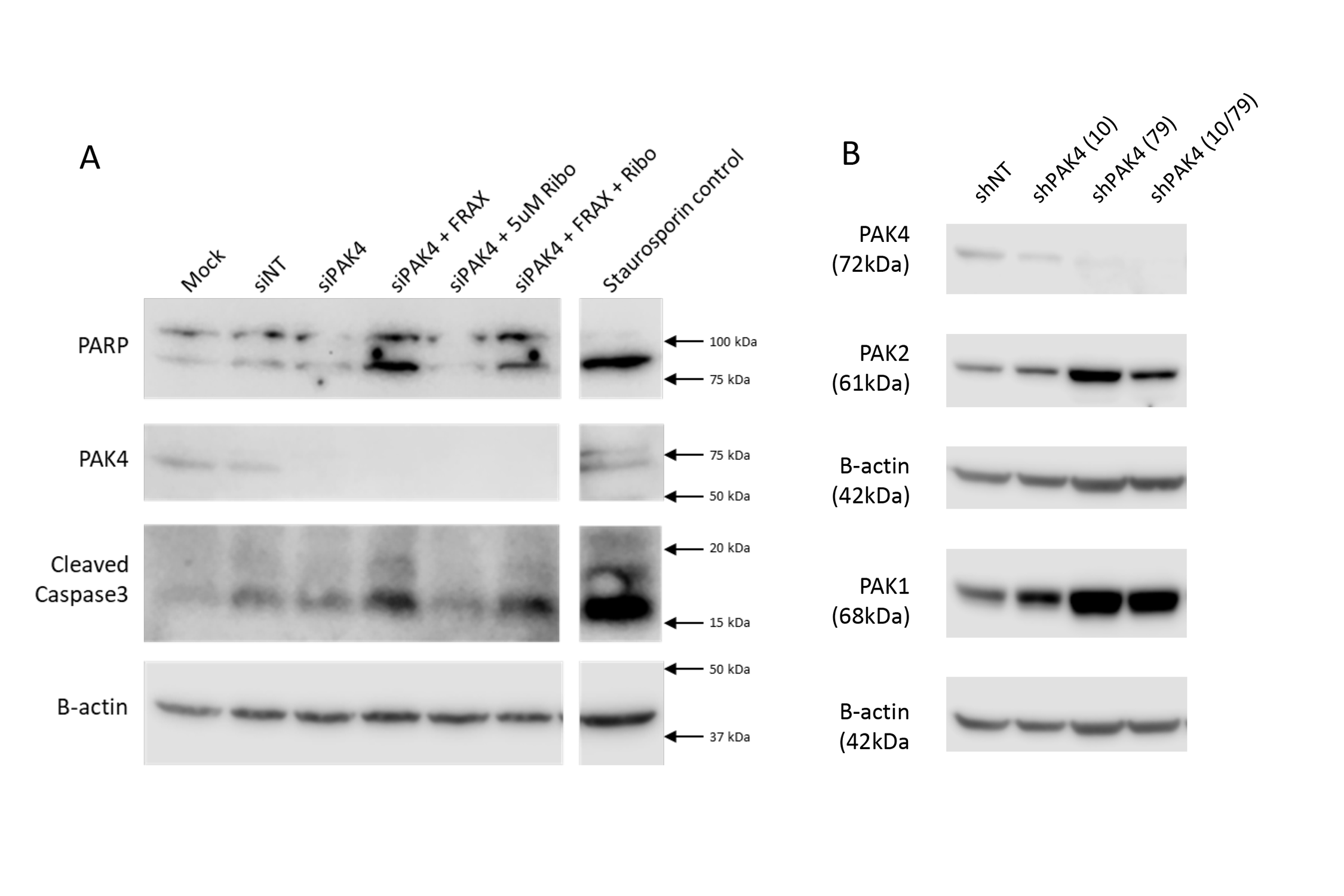

Supplement: S9 Fig — A. H322 cells treated with siNT (non-targeting) or siPAK4 for 72 hours (time needed for maximum PAK4 knock down) and FRAX486 and/or Ribociclib for the last 24 hours (treated at 48 hours). Apoptosis was measured by immunoblot detection of cleaved Caspase3 and PARP. B. Immunoblot showing the effect of PAK4 knock down on PAK1 and PAK2 protein expression, using H322 shNT, shPAK4(79) or shPAK4(10) alone, or a combination of shPAK4(79) and (10). (TIF) [file pone.0252927.s009.tif]
