# Supplementary material for: CDK4/6 inhibition synergizes with inhibition of P21-Activated Kinases (PAKs) in lung cancer cell lines
Source: PLoS One. 2021 Jun 17;16(6):e0252927. doi: 10.1371/journal.pone.0252927 (PMC8211232; doi:10.1371/journal.pone.0252927)
Supplement: S1 Raw images — (PDF) [file pone.0252927.s012.pdf]

CDK4/6 inhibition synergizes with inhibition of P21-Activated Kinases (PAKs) in lung cancer cell lines

Annotated Original Images

Gabriela M Wright, Nick T Gimbrone, Bhaswati Sarcar, Trent R Percy, Edna R Gordián, Fumi Kinose, Natália J Sumi, Uwe Rix, W Doug Cress

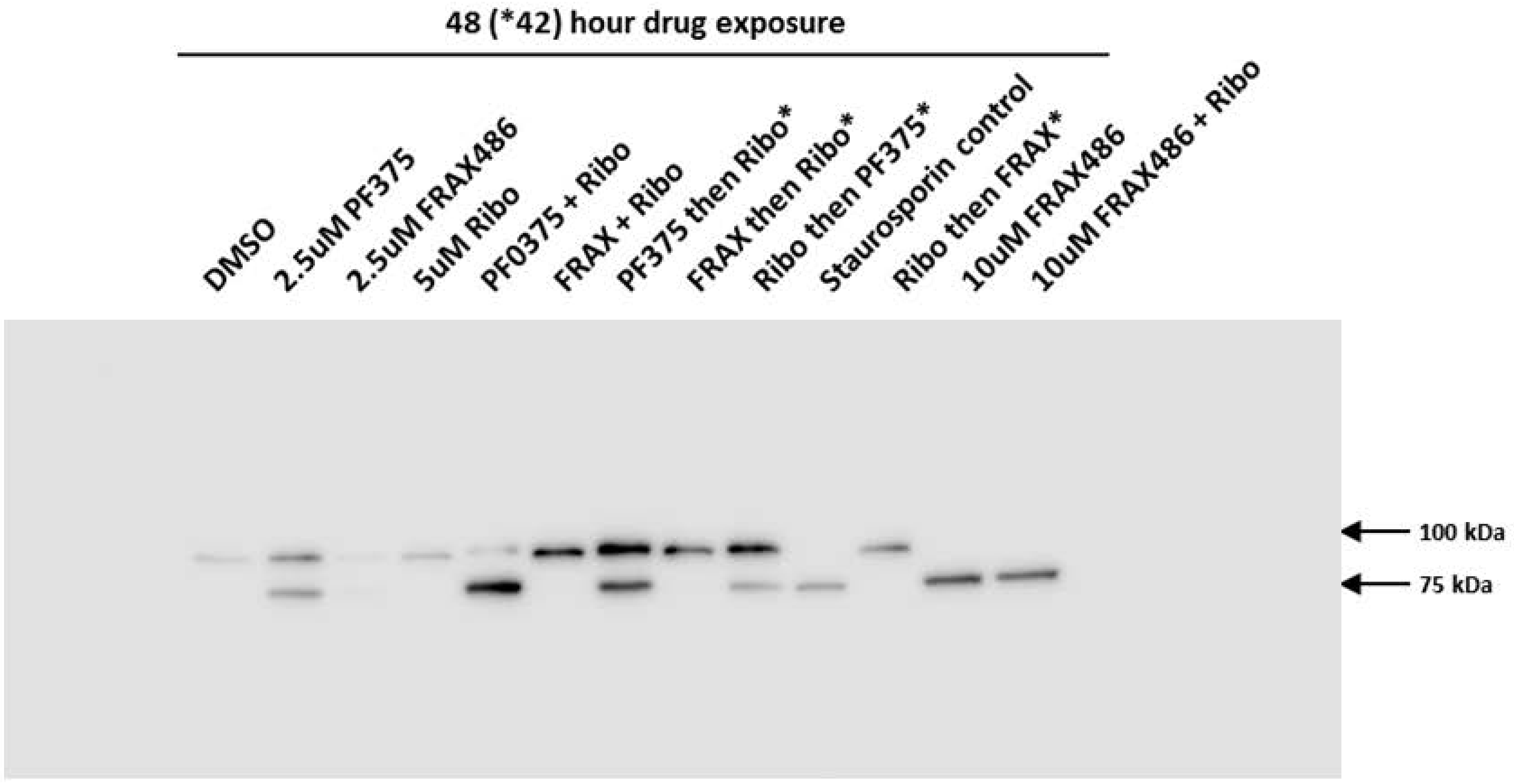

Figure 3, PARP panel, captured with LI-COR device and LI-COR Image Studio, Relevant MW markers are indicated manually since they are not detected under the imaging conditions. Additional details are in the main manuscript.

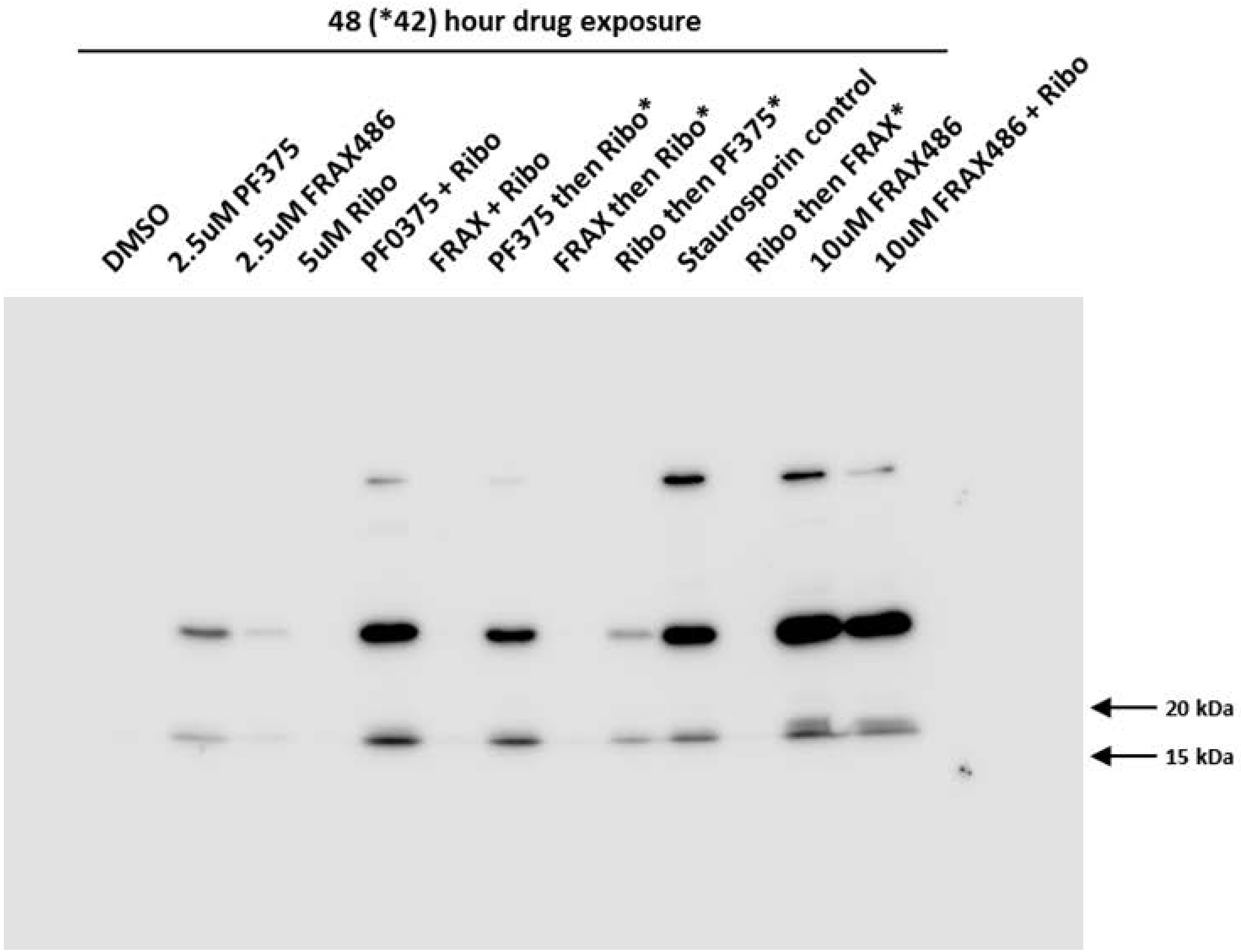

Figure 3, Cleaved Caspase-3 panel, captured with LI-COR device and LI-COR Image Studio, relevant MW markers are indicated manually since they are not detected under the imaging conditions

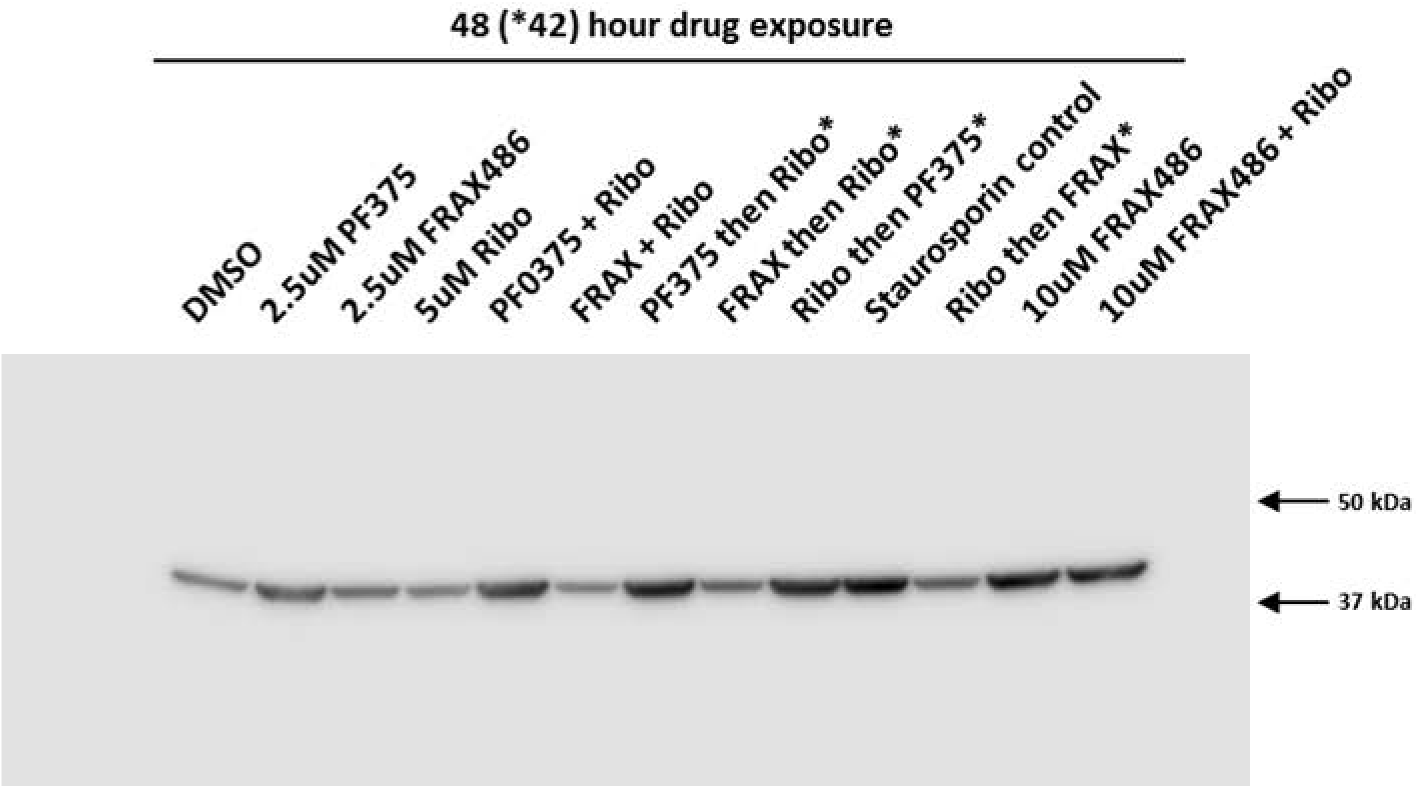

Figure 3, Beta-actin control panel, captured with LI-COR device and LI-COR Image Studio, relevant MW markers are indicated manually since they are not detected under the imaging conditions

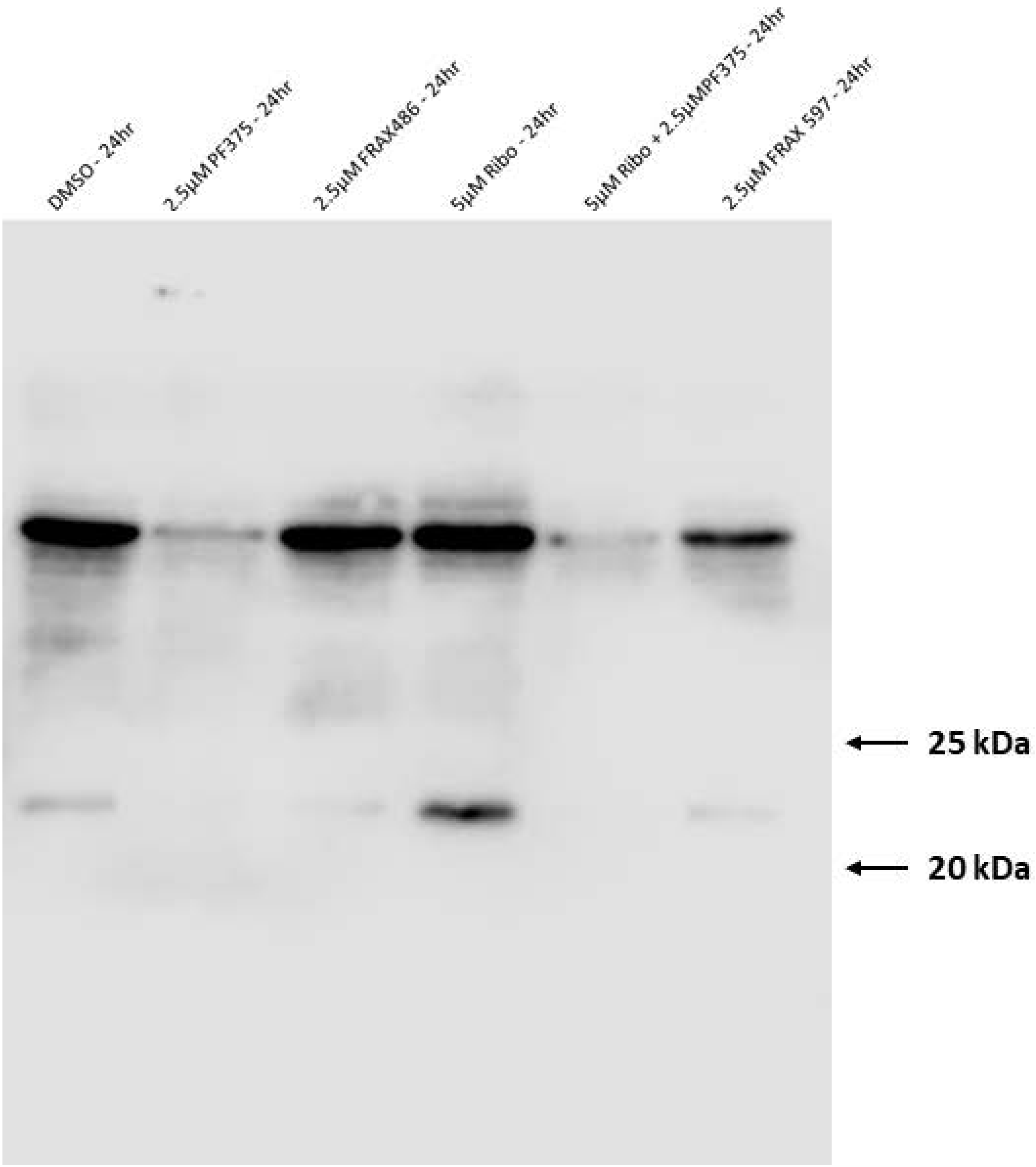

Figure S5A, Phospho-serine 112-BAD panel, captured with LI-COR device and LI-COR Image Studio, Relevant MW markers are not detected under the imaging conditions and are indicated manually.

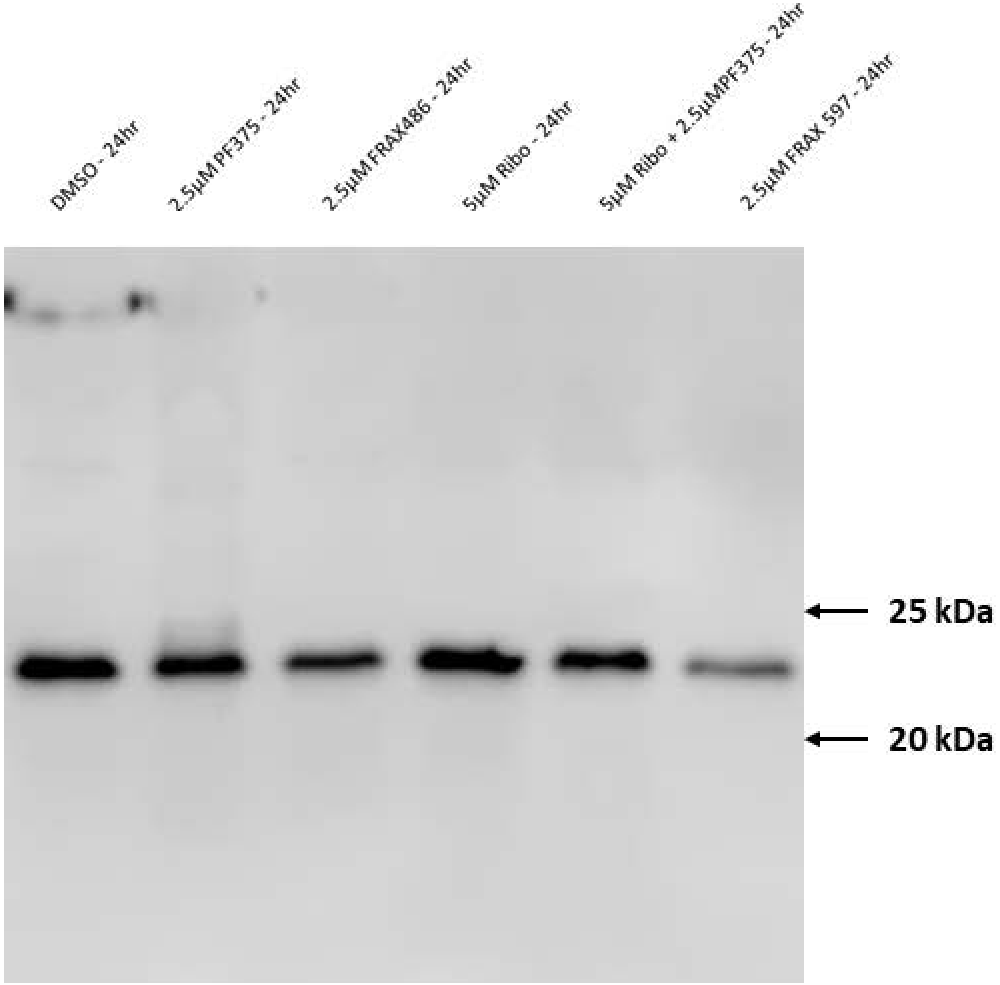

Figure S5A, BAD (23kDa), captured with LI-COR device and LI-COR Image Studio, MW markers are not detected under the imaging conditions

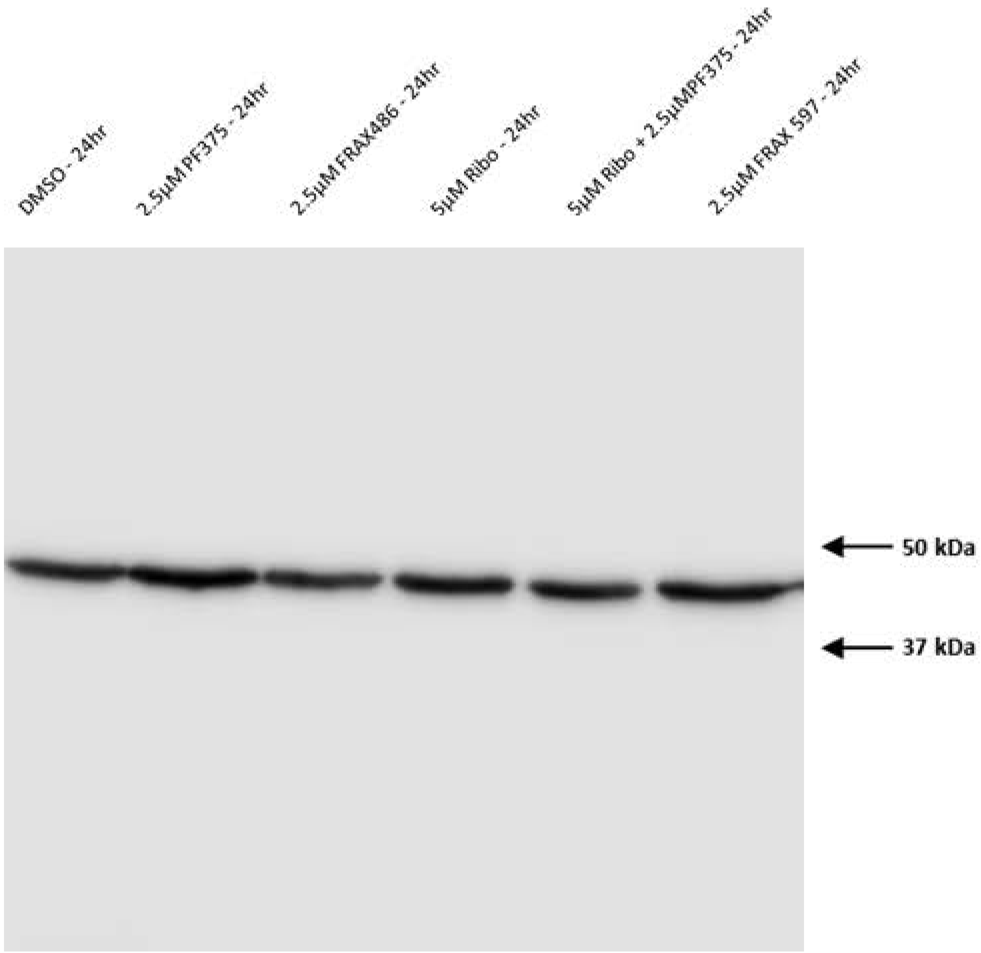

Figure S5A, B-actin panel, captured with LI-COR device and LI-COR Image Studio, MW markers are not detected under the imaging conditions, but positions are indicated

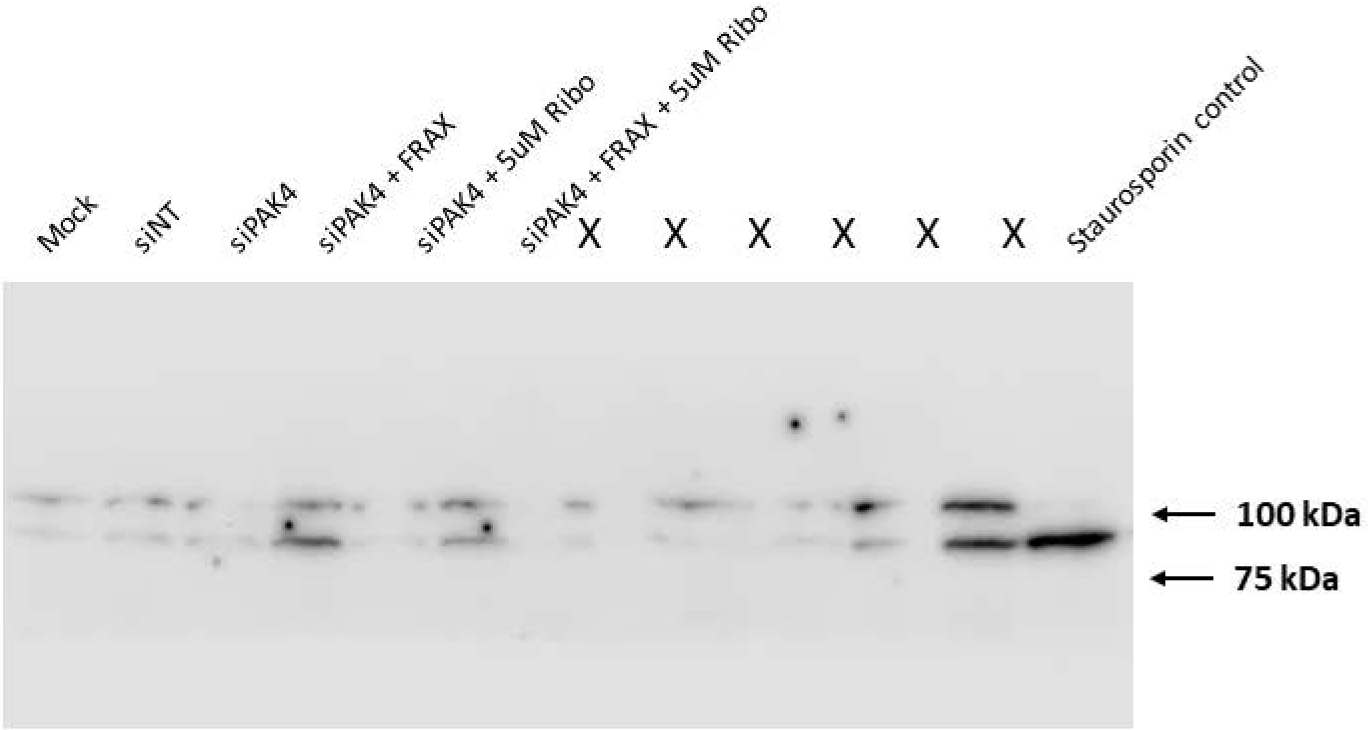

Figure S9A, PARP panel, captured with LI-COR device and LI-COR Image Studio, MW markers are not detected under the imaging conditions, but positions are indicated. X marks lanes that included irrelevant samples that were excluded from the final figure.

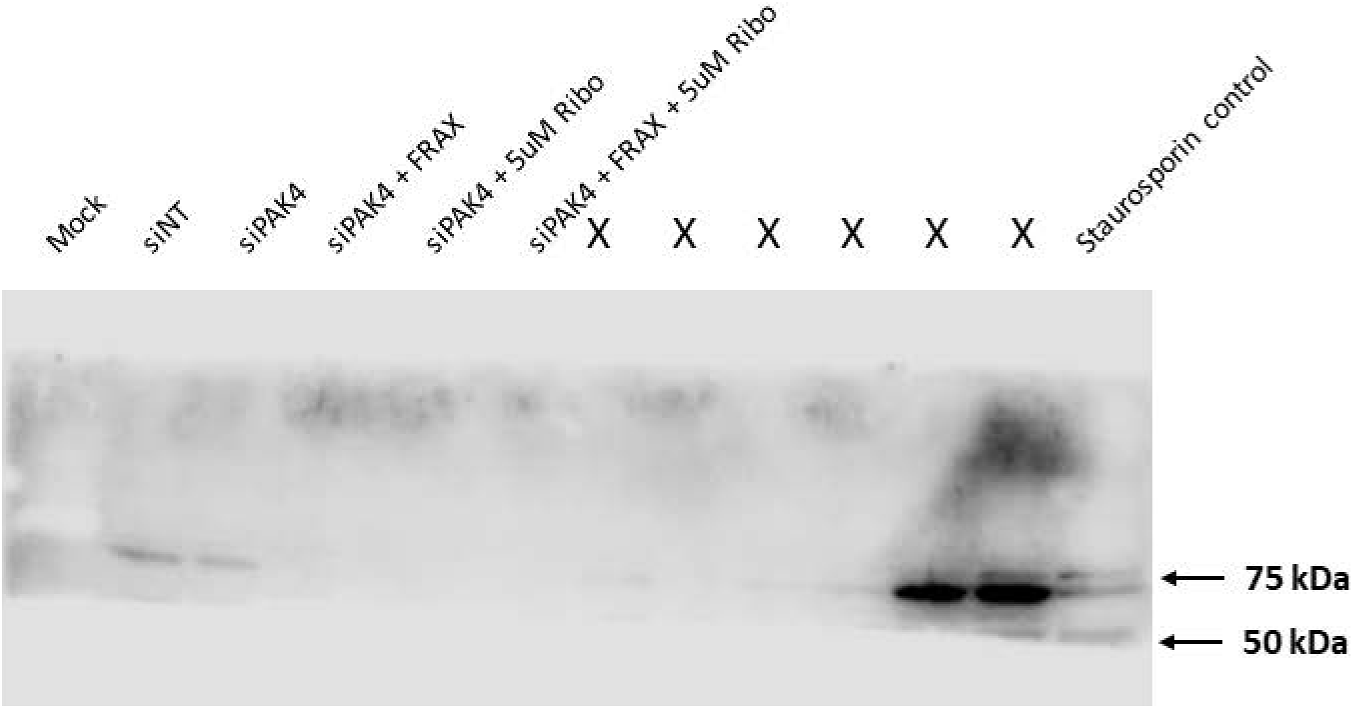

Figure S9A, PAK4 panel, captured with LI-COR device and LI-COR Image Studio, MW markers are not detected under the imaging conditions, but positions are indicated. X marks lanes that included irrelevant samples that were excluded from the final figure.

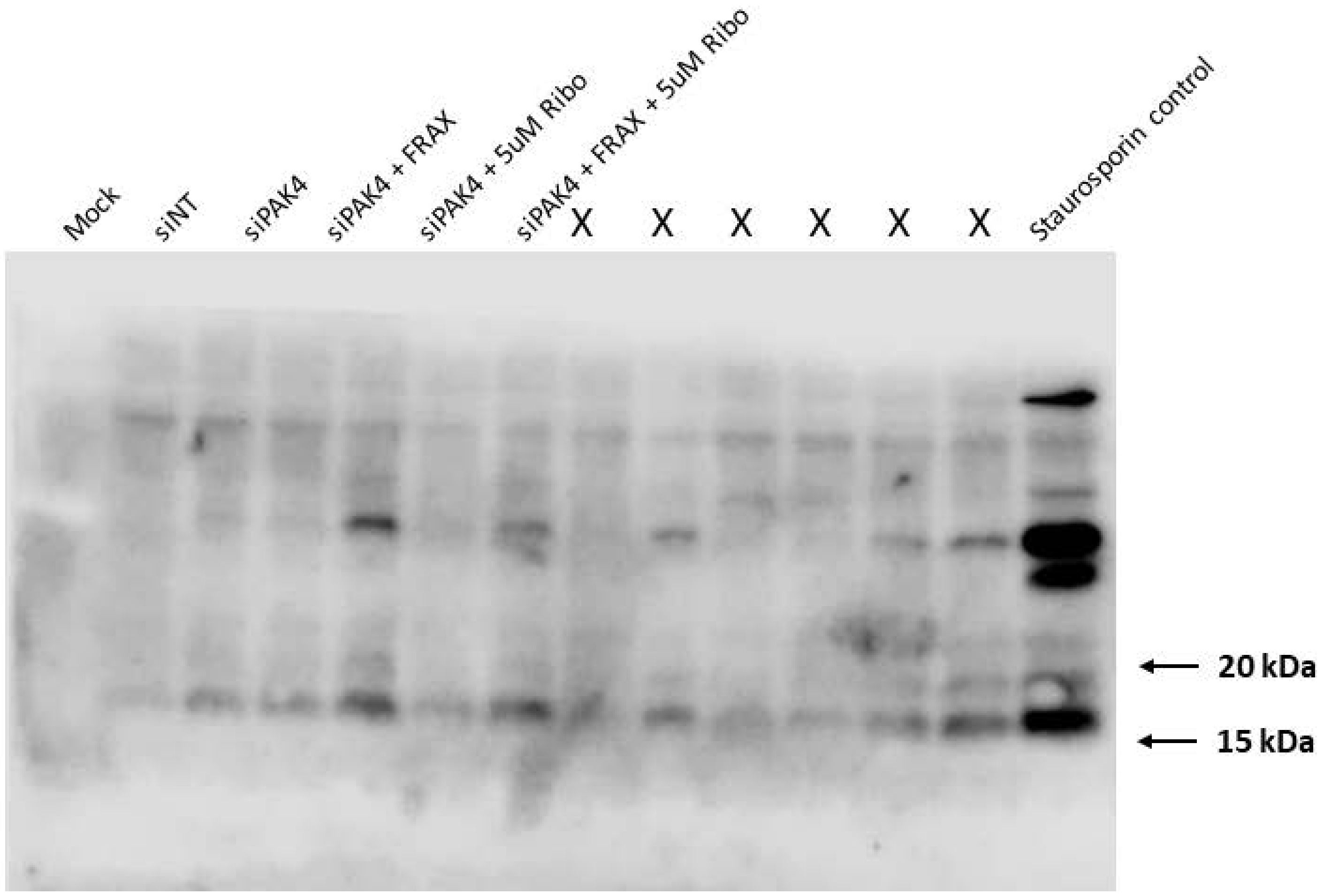

Figure S9A, Cleaved Caspase3 panel, captured with LI-COR device and LI-COR Image Studio, MW markers are not detected under the imaging conditions, but positions are indicated. X marks lanes that included irrelevant samples that were excluded from the final figure.

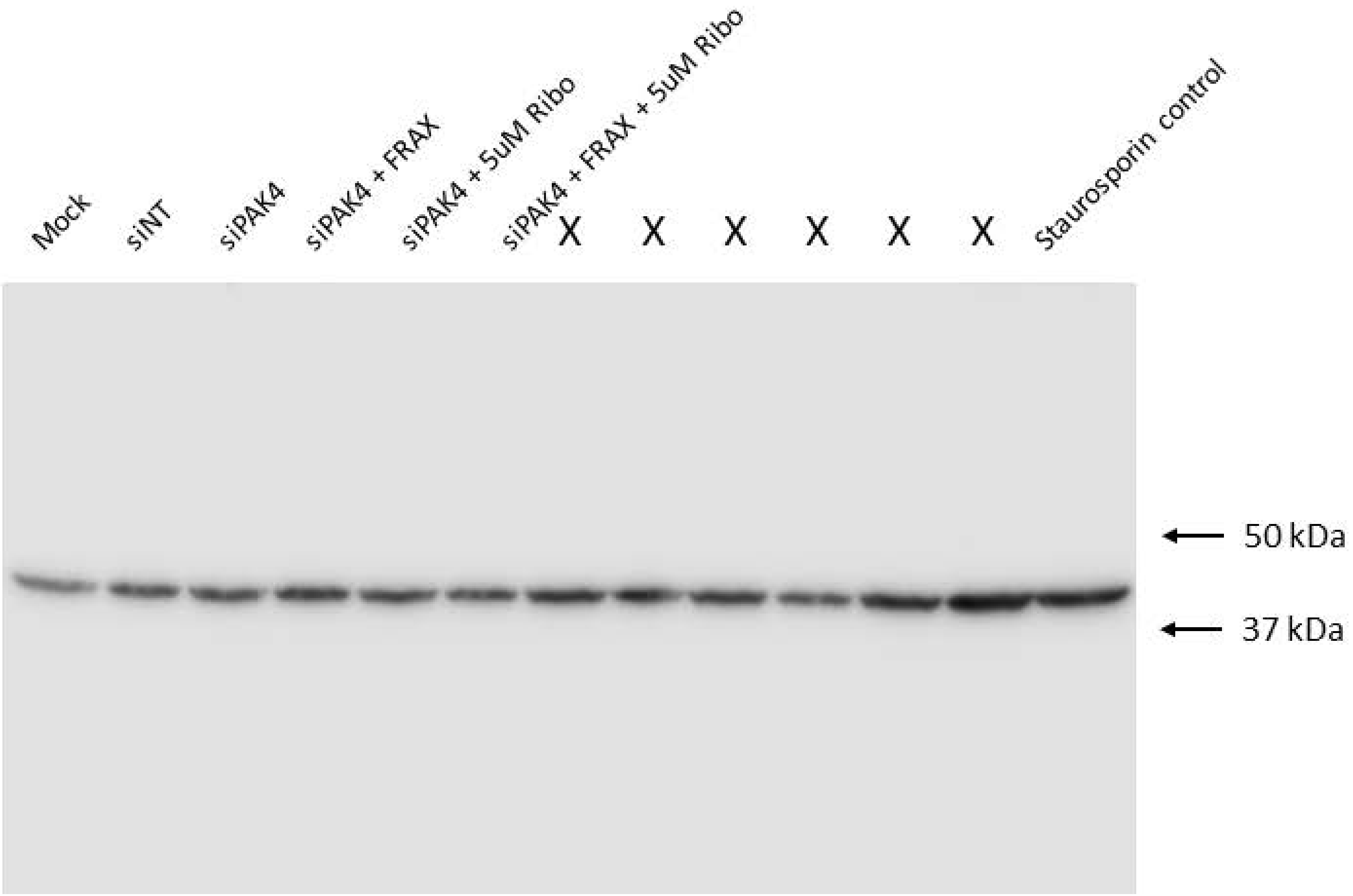

Figure S9A, B-actin panel, captured with LI-COR device and LI-COR Image Studio, MW markers are not detected under the imaging conditions, but positions are indicated. X marks lanes that were excluded from the final figure.

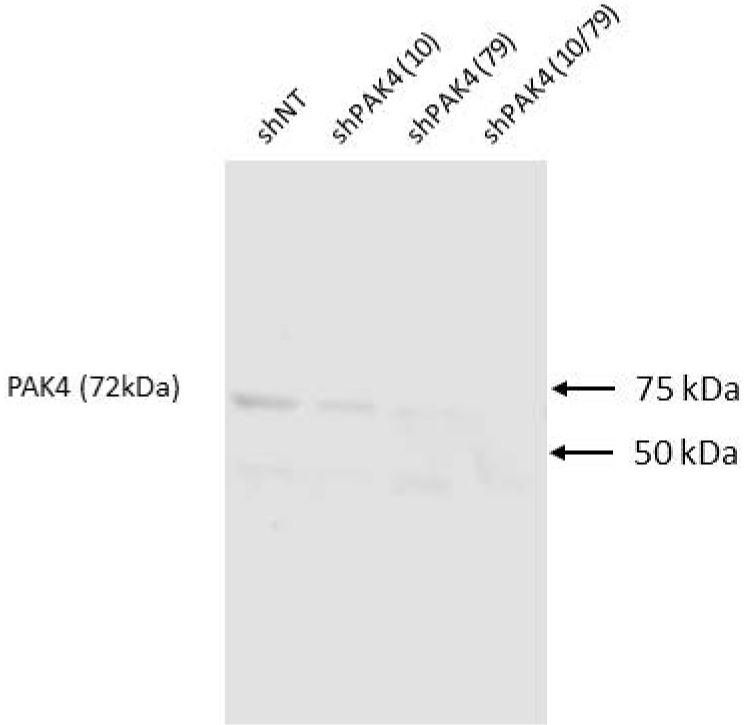

Figure S9B, PAK4 (72kDa) panel, captured with LI-COR device and LI-COR Image Studio, MW markers are not detected under the imaging conditions, but positions are indicated. X marks lanes that included irrelevant samples that were excluded from the final figure.

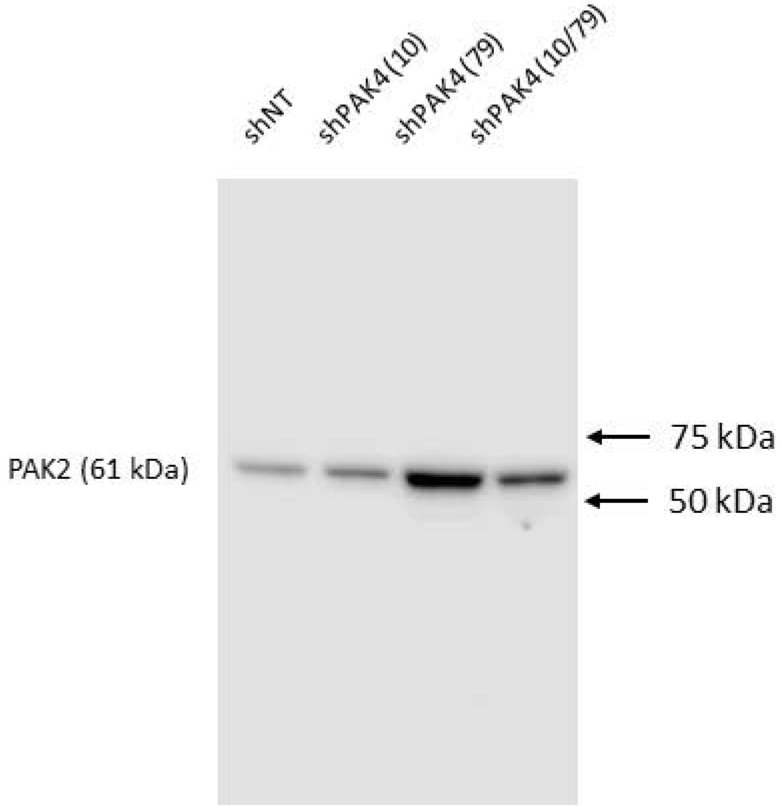

Figure S9B, PAK2 (61 kDa) panel, captured with LI-COR device and LI-COR Image Studio, MW markers are not detected under the imaging conditions, but positions are indicated.

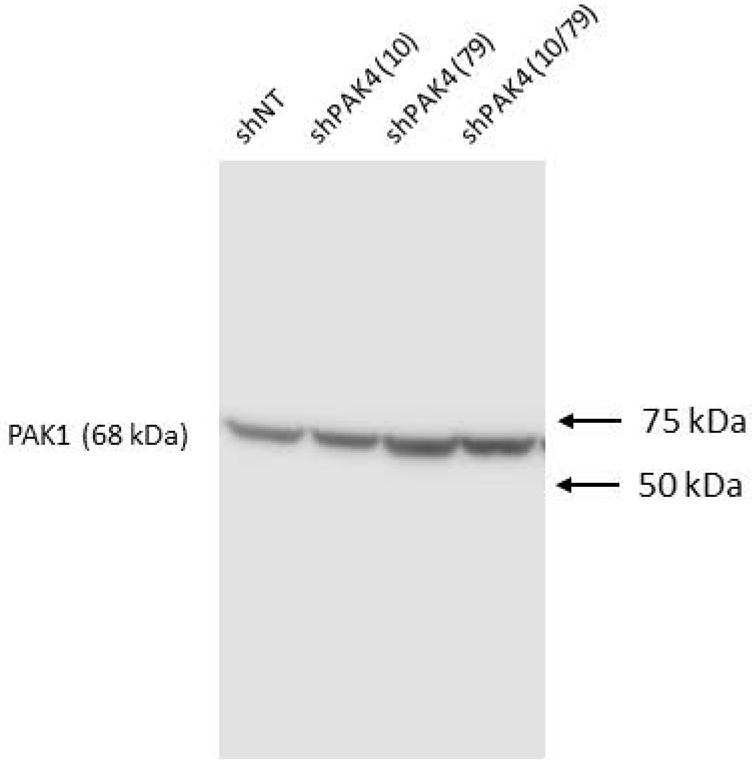

Figure S9B, PAK1(68 kDa) panel, captured with LI-COR device and LI-COR Image Studio, MW markers are not detected under the imaging conditions, but positions are indicated.

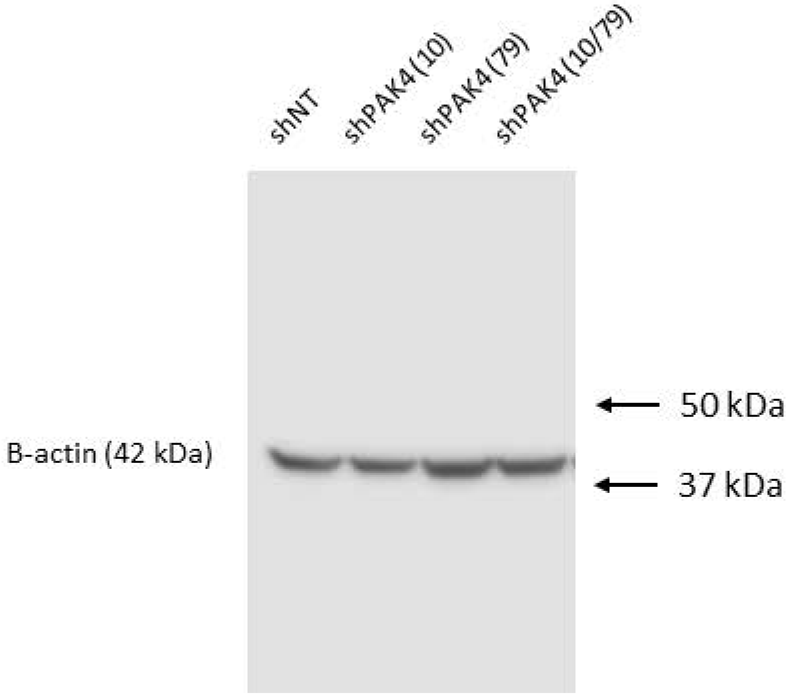

Figure S9B, B-actin lower band (42 kDa) panel, captured with LI-COR device and LI-COR Image Studio, MW markers are not detected under the imaging conditions, but positions are indicated.

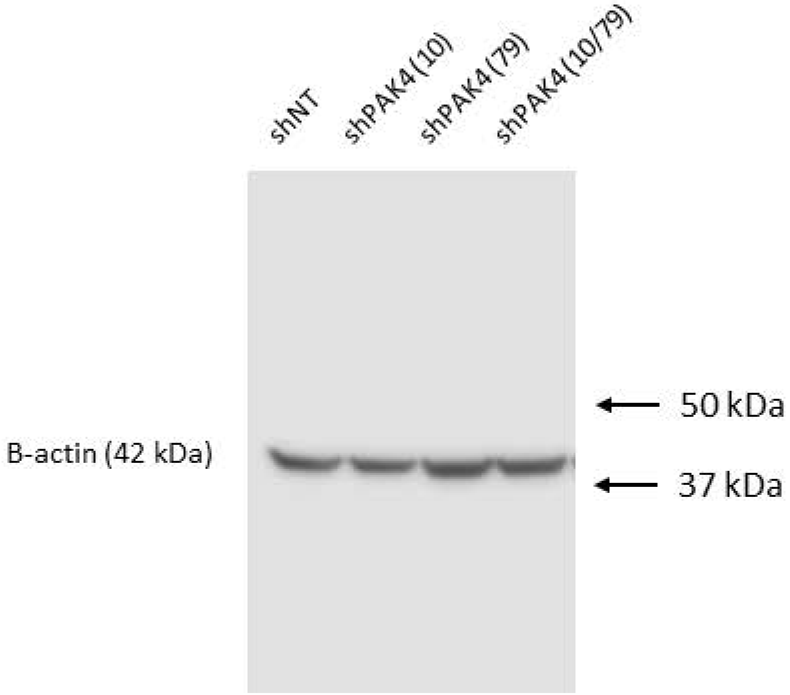

Figure S9B, B-actin lower band (42 kDa) panel, captured with LI-COR device and LI-COR Image Studio, MW markers are not detected under the imaging conditions, but positions are indicated.
